# A Random Particle Swarm Optimization Based on Cosine Similarity for Global Optimization and Classification Problems

**DOI:** 10.3390/biomimetics9040204

**Published:** 2024-03-28

**Authors:** Yujia Liu, Yuan Zeng, Rui Li, Xingyun Zhu, Yuemai Zhang, Weijie Li, Taiyong Li, Donglin Zhu, Gangqiang Hu

**Affiliations:** 1 School of Intelligent Manufacturing Engineering, Jiangxi College of Application Science and Technology, Nanchang 330000, China; 2 School of Computer Science and Technology, Zhejiang Normal University, Jinhua 321004, China; 3 School of Computing and Artificial Intelligence, Southwestern University of Finance and Economics, Chengdu 611130, China; litaiyong@gmail.com

**Keywords:** global optimization, particle swarm optimization, cosine similarity, classification

## Abstract

In today’s fast-paced and ever-changing environment, the need for algorithms with enhanced global optimization capability has become increasingly crucial due to the emergence of a wide range of optimization problems. To tackle this issue, we present a new algorithm called Random Particle Swarm Optimization (RPSO) based on cosine similarity. RPSO is evaluated using both the IEEE Congress on Evolutionary Computation (CEC) 2022 test dataset and Convolutional Neural Network (CNN) classification experiments. The RPSO algorithm builds upon the traditional PSO algorithm by incorporating several key enhancements. Firstly, the parameter selection is adapted and a mechanism called Random Contrastive Interaction (RCI) is introduced. This mechanism fosters information exchange among particles, thereby improving the ability of the algorithm to explore the search space more effectively. Secondly, quadratic interpolation (QI) is incorporated to boost the local search efficiency of the algorithm. RPSO utilizes cosine similarity for the selection of both QI and RCI, dynamically updating population information to steer the algorithm towards optimal solutions. In the evaluation using the CEC 2022 test dataset, RPSO is compared with recent variations of Particle Swarm Optimization (PSO) and top algorithms in the CEC community. The results highlight the strong competitiveness and advantages of RPSO, validating its effectiveness in tackling global optimization tasks. Additionally, in the classification experiments with optimizing CNNs for medical images, RPSO demonstrated stability and accuracy comparable to other algorithms and variants. This further confirms the value and utility of RPSO in improving the performance of CNN classification tasks.

## 1. Introduction

Scientific and technological development generate a significant amount of data, including medical data, transaction data, house-price data, and more. Researchers can utilize this data to enhance their understanding of convenient technology. However, dealing with diverse types of data requires the construction of complex models, which are processed through machine learning. The diversity of data poses limitations on the machine learning approach, as the parameters in machine learning need to be updated differently depending on the data being processed. Consequently, optimizing machine learning becomes a major issue.

As an optimization technique, meta-heuristic algorithms are driven by natural phenomena and behaviors [1]. These algorithms possess the benefit of not requiring specialized understanding regarding the issue at hand. Instead, they iteratively explore and solve problems in a certain space, providing robustness and universality [2]. Particle Swarm Optimization (PSO) [3], a type of meta-heuristic algorithm, is representative and widely used. PSO is an optimization technique that mimics the natural hunting behavior of birds. It is characterized by its simplicity, requiring fewer parameters and being easy to implement compared to other methods. PSO exhibits good learning ability through the interaction of individual information during the evolutionary process. Consequently, it effectively addresses numerous real-world issues, including energy systems [4], path planning [5], image processing [6], DNA computing [7], and engineering optimization [8]. Consequently, there is significant research interest in combining PSO with machine learning techniques. In summary, meta-heuristic algorithms, such as PSO, offer a powerful approach for solving optimization problems. Their ability to adapt and explore solution spaces without requiring explicit problem knowledge makes them valuable tools for a wide range of applications.

PSO has gained significant attention in recent research for its ability to solve challenging problems in machine learning. Several studies have applied PSO to optimize various machine learning models and algorithms. For instance, Yong Zhu improved the LeNet-5 model by employing PSO to optimize its parameters [9]. Wu Deng optimized the parameters of the Least Squares Support Vector Machine (LS-SVM) for fault classification using an enhanced PSO algorithm [10]. Rajesh K. Yadav and Anubhav introduced a hybrid training algorithm combining PSO and the genetic algorithm (GA) alongside Adam Optimization to enhance the training process of artificial neural networks [11]. Kun Zhang utilized a PSO-optimized Least Squares Support Vector Machine (LSSVM) to establish a fault diagnosis model [12]. J. Beschi Raja and S. Chenthur Pandian employed PSO to optimize the Fuzzy Clustering Mean (FCM) for disease prediction [13]. Mohammed Nasser Al-Andoli integrated PSO with the backpropagation (BP) algorithm for malware detection [14]. Chao Huang used PSO to optimize Deep Convolutional Neural Networks (DCNNs) for automatic defect detection and classification [15]. Thomas George employed PSO for feature selection in electroencephalogram (EEG) signal classification [16]. Mohamed Issa combined PSO with the bat optimization algorithm for improved detection techniques [17]. Abhishek Dixit utilized a mix of algorithms, primarily based on the Differential Evolutionary Algorithm (DE) and PSO, for optimizing feature selection and classification [18]. Muhammad Sharif employed PSO for image segmentation and the GA for feature selection [19]. Hanuman optimized the FCM clustering algorithm for medical image segmentation using PSO [20]. Debendra Muduli leveraged Multi-Objective Discrete Particle Swarm Optimization (MODPSO) to enhance the performance of feedforward neural networks in breast cancer detection by optimizing hidden node parameters [21]. However, traditional PSO approaches face challenges in complex optimization models, as they can easily become caught in local optima, resulting in premature convergence. Additionally, the stability of the optimization capability of PSO leaves room for improvement [22].

To further boost the optimization capability of PSO, this article proposes a random particle swarm optimization algorithm based on cosine similarity, referred to as RPSO. RPSO adjusts the internal parameters of PSO and introduces the Random Contrastive Interaction mechanism and quadratic interpolation (QI). It employs cosine similarity to choose between the two mechanisms, which efficiently and dynamically updates population information, enhancing the global and local optimization capabilities of the algorithm. The performance tests were carried out based on two dimensions of CEC 2022 and compared with the PSO variant and the top algorithm of CEC to verify the competitiveness and effectiveness of RPSO. Finally, RPSO was optimized for the hyperparameters of the CNN, and the optimized model was confirmed to possess superior diagnostic abilities in both datasets. The specific work performed and innovations produced in this study are as follows:

- Introduction of the Random Contrastive Interaction mechanism.
- Introduction of the Quadratic interpolation mechanism.
- Design of a judgment condition based on cosine similarity.
- Comparison of PSO variants and the top algorithm in two dimensions of CEC 2022.
- Optimization of CNN for the diagnosis of two diseases.

This paper is structured as follows: Section 2 introduces the theory of PSO and cosine similarity; Section 3 provides the introduction and analysis of the proposed algorithm; Section 5 presents the testing of the proposed algorithm based on CEC 2022; Section 4 highlights the improvement provided by the suggested algorithm when applied to CNN for classification experiments; and the last section concludes the work of the paper with a summary and discussion.

## 2. Related Theories

### 2.1. Particle Swarm Algorithms

In the PSO algorithm optimization process, the position information vector x is symbolized by a matrix of *N* × *d* and $x_{i}=(x_{i1},{\;x}_{i2},\cdots ,x_{id})$, and the generated velocity vector $v_{i}=(v_{i1},v_{i2},\cdots ,v_{id})$. The velocity and position are continuously revised through the following equation [12].

(1) $$v_{i}(t+1)=wv_{i}(t)+z_{1}r_{1}(x_{pb,i}(t)-x_{i}(t))+z_{2}r_{2}(x_{gb,i}(t)-x_{i}(t))\;x_{i}(t+1)=x_{i}(t)+v_{i}(t+1)$$

In Equation (1), $v_{i}(t+1)$ and $x_{i}(t+1)$ represent individual i’s velocity and position during generation *t*, *z*_1_ and *z*_2_ denote the two acceleration coefficients, *r*_1_ and *r*_2_ denote the uniform random numbers [0, 1], and *w* is the inertia weight. $x_{pb}(t)$ and $x_{gb}(t)$ denote the global optimum and the historical optimal position of an individual at generation *t*, respectively.

### 2.2. Cosine Similarity

The cosine similarity measure serves to assess how similar two vectors are by finding the cosine of their intersection angle [23]. This value ranges between −1 and 1, where a cosine similarity value of 1 indicates that the vectors share the same direction, 0 indicates orthogonality (a 90-degree angle between the vectors), and −1 indicates that the vectors share opposite directions.

**Figure 1:**
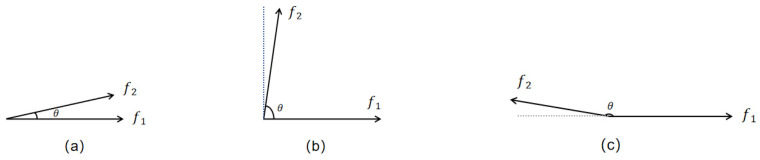
Angles in 2D space.

Unlike other similarity measures, cosine similarity disregards vector lengths and solely examines their pointing directions. As a result, it can determine whether two vectors are essentially heading in the same direction based solely on the cosine of the angle between them. Cosine similarity is typically utilized for positive spaces and yields values within the range [−1, 1]. This is illustrated in Figure 1, which represents the most common 2D space.

Figure 1a demonstrates that as the angle between two vectors approaches 0°, the cosine value approaches 1, indicating a high similarity in direction. In Figure 1b, a cosine value close to 0 indicates that the vectors are orthogonal, meaning they are independent of each other. Figure 1c illustrates that a cosine value closer to −1 implies that the vectors are oriented in opposite directions.

It is important to note that cosine similarity is independent of vector length and only considers the direction of the vectors. The formula for calculating cosine similarity is as follows [24]:

(2) $$\cos(\theta )=\frac{\sum_{i=1}^{n}\left(A_{i}\times B_{i}\right)}{\sqrt{\sum_{i=1}^{n}{\left(A_{i}\right)}^{2}\times \sqrt{\sum_{i=1}^{n}{\left(B_{i}\right)}^{2}}}}$$

where $A_{i}$ and $B_{i}$ are the *i*-th components of *A* and *B*, respectively.

## 3. The Proposed Algorithm

This subsection provides a description of the proposed algorithm and its principles.

### 3.1. Related Research from PSO

In recent years, researchers have made significant advancements in improving the PSO, which can be broadly divided into four primary categories: hybrid algorithms, multiple populations or multi-sampling approaches, adaptive learning mechanisms, and other miscellaneous techniques.

Hybrid algorithms have gained popularity in enhancing the performance of PSO by combining different optimization algorithms. For instance, Zeng [25] incorporated the DE algorithm into PSO to address the issue of premature convergence. Zaman [26] fused the Backtracking Search Optimization Algorithm (BSA) with PSO by modifying the mutation and crossover operators using neighborhood information, resulting in an improved convergence rate. Xu [27] proposed a strategy learning framework that utilized an adaptive DE algorithm to learn an optimal combination of strategies. Taking inspiration from the bee-foraging search mechanism of the Artificial Bee Colony (ABC) algorithm, Chen [28] developed a hybrid algorithm to tackle complex global optimization problems (GOPs). Khan [29] combined the local search ability of the Gravitational Search Algorithm (GSA) with the social thinking capability (gbest) of PSO. Another approach was taken by Molaei [30], who combined PSO with the GA. Ahmed [31] proposed a novel Hybrid Particle Swarm Optimization and Genetic Algorithm (HPSOGA) to minimize the energy function of molecules. HPSOGA balances exploration and exploitation, enhances diversity, and avoids premature convergence. It outperforms standard PSO and benchmark algorithms in solving large-scale global optimization problems and molecular energy functions.

In the field of multiple population or multi-sample techniques, several approaches have been proposed to enhance Particle Swarm Optimization (PSO). Xia [32] developed a Triple Archives PSO (TAPSO) that incorporates three different types of archives: elite particles, profiteer particles, and outstanding exemplars. These archives guide future merit-seeking mechanisms through genetic properties. Zhang [33] introduced the Particle Swarm Optimization-Alternating Least Squares (PSO-ALS) algorithm, which adaptively groups the population into sub-swarms and further divides particles into ordinary particles and the locally best particle within each sub-swarm. Two different learning strategies are designed to update the particles. Li [34] divides the particle swarm into elite and normal groups based on a ranking system and defines different types of neighborhoods accordingly. Sheng [35] proposed a PSO variant with multi-level population sampling and dynamic p-learning mechanisms. Lu [36] proposed an Enhanced Multi-Swarm Cooperative Particle Swarm Optimizer (EMCPSO) that divides the entire population into four identical sub-swarms. This algorithm incorporates a delayed-activation (DA) mechanism to detect sub-swarm stagnation and introduces a repulsive mechanism to prevent premature clustering of the entire population.

In terms of adaptive learning mechanisms, Liu [37] developed a novel PSO algorithm that incorporates a sigmoid-function-based weighting strategy. This strategy adjusts the acceleration coefficients by considering the distance of particles to the global best position and their personal best position. Liu [38] also proposed several other adaptive mechanisms, including a chaos-based non-linear inertia weight, stochastic and mainstream learning strategies, an adaptive position updating strategy, and a terminal replacement mechanism. Li [39] introduced a landscape-based adaptive operator selection mechanism. This mechanism quantifies the landscape modality, selects the most suitable evolutionary operator based on the population’s needs at different evolutionary stages, and adopts the mirrored boundary handling method to alleviate trapping in local optima. Zhang [40] proposed a particle swarm optimization algorithm with an empirical balance strategy (EBPSO), which selects a better search strategy from two equations using an adaptive adjustment mechanism. The algorithm dynamically adjusts the influence weight of the search equations and introduces a dynamic random search mechanism. Wang [41] proposed a hybrid PSO called Adaptive Strategy PSO (ASPSO), which incorporates a chaotic map, a position updating strategy, elite and dimensional learning strategies, and a competitive substitution mechanism. Li [42] proposed a novel PSO variant with a state-based adaptive velocity limit strategy (PSO-SAVL). In this strategy, the velocity limit is adaptively adjusted according to the estimated evolutionary state (ESE), with high values representing the global searching state and low values representing the local searching state.

In addition to the aforementioned categories, there are several other methods for improving PSO. Wang [43] presented a reinforcement learning strategy to adaptively adjust the optimization mechanism of PSO. Tian [44] proposed Variable Surrogate Model-based Particle Swarm Optimization (VSMPSO), which utilizes a single surrogate model constructed through simple random sampling and a variable model management strategy. Liu [45] incorporated Evolutionary Game Theory (EGT) to control the population state and proposed a selection mechanism and a mutation mechanism inspired by EGT concepts. Liu [46] employed Gaussian white noise with adjustable intensity to randomly perturb the acceleration coefficients, aiming to explore the problem space more extensively. Machado [47] introduced the concepts of complex-order derivatives (CD) and conjugate-order differentials.

Despite achieving certain optimization results, the aforementioned research approaches still have limitations. For example, hybrid algorithms may struggle to guarantee the complementary advantages of the two algorithms. Multiple swarms may not ensure effective local development. Adaptive mechanisms may possess irreversible search mechanisms. Additionally, it can be challenging to ensure the learning and adaptive ability of the algorithm in the existing research. To solve these limitations, this paper proposes a judgment mechanism based on cosine similarity and introduces the Random Contrastive Interaction (RCI) and QI mechanisms to jointly enhance the effective search capability of the algorithm.

### 3.2. Random Contrastive Interaction

**Figure 2:**
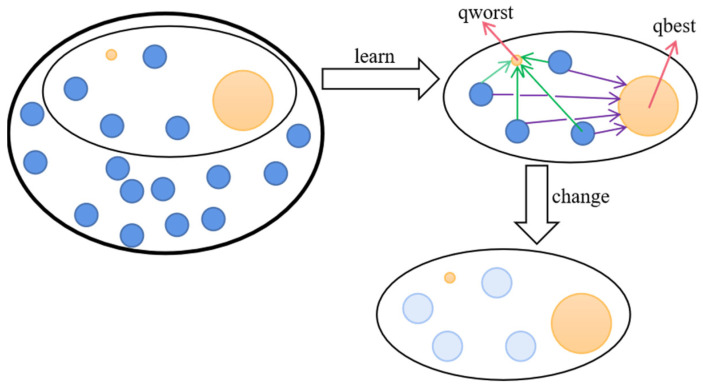
Schematic diagram of RCI.

In order to cope with complex and variable optimization environments, it is difficult for traditional particle swarms to maintain a good search ability to find high quality information, especially when the formulas use information about historical optimal positions. This can lead to a situation where multiple historical optimal positions cannot be updated with the global optimal position, resulting in a lack of diversity in the algorithm. For this reason, we introduce Random Contrastive Interaction [48], which provides better evolutionary information by means of the states within the population adaptively learning from each other. A specific schematic is shown in Figure 2.

As shown in Figure 2, P individuals are randomly selected from the population to form a random topology. Within this small population, there will be a local best solution, *qbest*, and a worst solution, *qworst*. The role of RCI is to enable the individuals within the small population to learn from *qbest* and *qworst*, and to interact with each other to obtain information, meaning that dynamically updating an individual’s position enhances the flexibility of the algorithm. The details are as follows [48]:

(3) $$Z_{i}=Q_{1}\cdot V_{i}+Q_{2}\cdot (qbest-x_{Pi})+{\varphi \cdot Q}_{3}\cdot (qworst-x_{Pi})\;S_{i}=x_{Pi}+Z_{i}$$

where, $Q_{1}$, $Q_{2}$, $Q_{3}$ are uniform random numbers in the range of [0, 1] and φ is the learning parameter controlling *qworst*, which is 0.3. An optimal setting of ϕ for RPSO falls within a moderate range, neither too large (such as φ = 0.5) nor too small (such as φ < 0.3), as extremes hinder RPSO from achieving optimal performance. A φ that is too small diminishes the impact of the worst position on each updated particle, potentially causing rapid movement of particles towards the location of the best position and resulting in premature convergence or being trapped in local optima. On the other hand, a φ that is too large amplifies the influence of the worst position excessively, disrupting swarm convergence and leading to suboptimal performance of RPSO. $Z_{i}$ is the velocity of the newly generated individual and $S_{i}$ is the position of the newly generated individual. $x_{Pi}$ denotes the position of the ith individual in the selected population *P*. It can be seen that the selection of individuals makes it possible to continuously move from the locally optimal individual to the locally worst position, increasing the exchange of information in the population and also improving the global exploration capacity.

**Figure 3:**
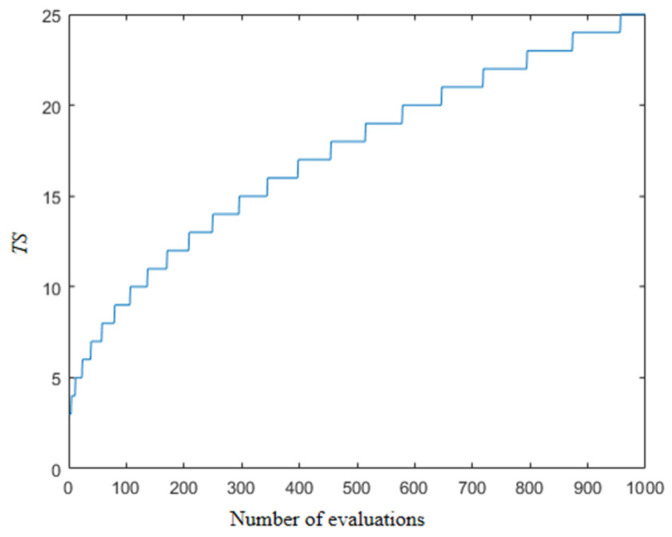
*TS* in literature [48].

Which the number of randomly selected individuals is constantly changing, in the early stage of the algorithm, in the stage of global exploration needs global search capability, at this time a larger number of individuals need to RCI operation, and in the later stage, a portion of the individuals need to carry out a certain amount of exploration to increase the diversity of the population. In the literature [48], the variation in the size of the selected populations is shown in Figure 3. *TS* is the population size for each selection. As shown in the Figure 3, the randomly selected population size presents an increasing manner, which will lead to a problem that the local exploitation ability is very poor at the later stage, and the feasible solution cannot be mined effectively. Therefore, this paper designs a new population design scheme, which is formulated as follows:

(4) $$TQ=(rand(1)+1)\times (1-t/T)\times N/2\;{TS}_{t}=round(max(TQ,N/10))$$

**Figure 4:**
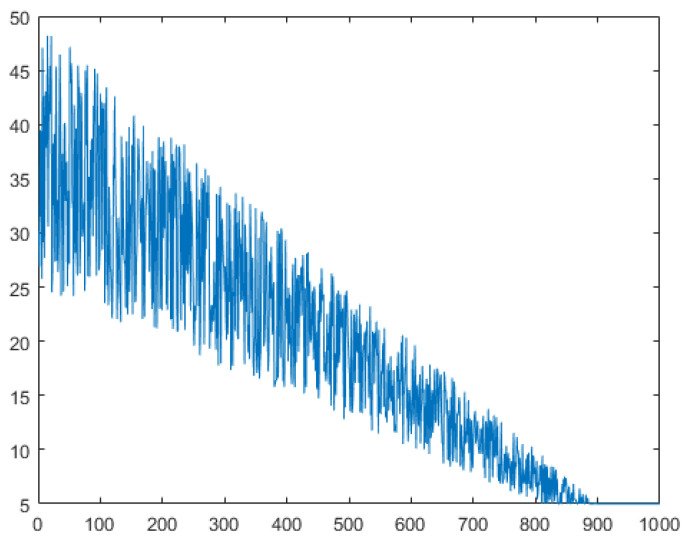
Proposed *TS*.

The use of a max function is needed to ensure that there are at least *N*/10 individuals in the population. This ensures that the individuals need to carry out a certain exploration, in which the value of *TQ* is constantly changing. The overall trend is gradually decreasing, but this is an oscillating change. Taking the population number of 50 as an example, the specific value changes as shown in Figure 4; it can be seen that the range of changes in the early stage is larger, and in the later stage, this value gradually becomes smaller, and finally the structure of *N*/10 is unanimously adopted. Such a screening mechanism not only ensures the diversity of the population but also enables a detailed search of a certain area to find a higher quality of feasible method. On the other hand, the oscillation characteristics are beneficial, mainly reflected in the reversible changes in the population size. This can diversify the search process and compensate for any vulnerabilities in the algorithm’s search. The specific pseudo-code is shown in Algorithm 1.
**Algorithm 1.** Random Contrastive Interaction (RCI)***Input:****t: global optimum position**T: Matrix of population information**N: population size****fit:*** *Matrix of objective function valuesx: Position of the population****Output:***X*: Matrix of newly generated stock information*The number of individuals TS to be selected for this iteration is calculated according to Equation (4);**1.*      ***rs*** *=* *randperm(N, TS); %Generate non-repeating individual subscripts based on TS.**2.*      *[**af**, **bf**] = sort(**fit**); % af is the result of the ascending order of the fitness values and bf is the corresponding subscript position.**3.*      *db = find(**bf** == **rs**(1));Find the location of the optimal individual in rs.**4.*      *dw = find(**bf** == **rs**(TS));Find the worst individual position in rs.**5.*      ***dbest**** = **pos**(bbb(db),:);**6.*      ***dworst**** = **pos**(bbb(dw),:);**7.*      ***For*** *i = 1:size(**rs**,2)**8.*          ***For*** *j = 1:size(**x**,2)**9.*              *Calculate the corresponding new position in rs according to Equation (3).**10.*          ***End****11.*      ***End****Return* X^*^


### 3.3. Quadratic Interpolation (QI)

QI is a better local search technique that employs a parabola to adapt the form of a quadratic function to locate the extreme points of a curve [49]. The quadratic interpolation technique is used to further accelerate the information exchange of the population and improve the local search ability. The specific formula is as follows [50]:

(5) $$x_{new}=0.5\cdot (\frac{\left(x_{1}^{2}-x_{2}^{2})\cdot f(x_{3})+(x_{3}^{2}-x_{1}^{2})\cdot f(x_{1})+(x_{1}^{2}-x_{3}^{2})\cdot f(x_{2}\right)}{\left(x_{1}-x_{2})\cdot f(x_{3})+(x_{2}-x_{3})\cdot f(x_{1})+(x_{1}-x_{3})\cdot f(x_{2}\right)})$$

where *x*_1_, *x*_2_, and *x*_3_ are three individuals randomly selected from the population. In this paper, *x*_1_ represents the location of the globally optimal individual, while *x*_2_ and *x*_3_ represent two randomly selected individuals from the population. Algorithm 2 provides the specific pseudo-code. By facilitating information interaction between the globally optimal individual and the two individuals from the population, the algorithm guides the population to converge towards the vicinity of the optimal solution, thereby enhancing the algorithm’s local search capability.
**Algorithm 2.** Quadratic interpolation (QI)***Input:******gbest****: global optimum position****x****: Matrix of population information****Output:***x^*^: Matrix of newly generated stock information*1.*      
***For***
* i = 1:size(x)*
*2.*      
***k***
* = randperm(N, 2)%Two non-repeating individuals were randomly generated.*
*3.*      
*The positional information of the new individual is calculated by substituting the three individuals **k**(1), **k**(2), and **gbest** into Equation (5).*
*4.*      
***End***
Return x^*^

### 3.4. Setting of Parameters

In the particle swarm algorithm, with fixed parameters, there is a fixed search amplitude and it is difficult to increase the diversity. Many scholars have proposed using the following formula for the setting of *z*_1_ and *z*_2_ with weights w [51].

(6) $$w=w_{max}-(w_{max}-w_{min})\times t/T\;z_{1}=2.5-2\times t/T\;z_{2}=0.5+2\times t/T$$

where $w_{max}$ is 0.9 and $w_{min}$ is 0.2, w decreases with the number of iterations, and the population’s diversity levels are gradually reducing. $z_{1}$ and $z_{2}$ changes are set by biasing the historical optimum and the global optimum; in the early stage of the algorithm, a larger $z_{1}$ and a smaller $z_{2}$ cause the historical optimum position to have a larger bias, which can cause the global exploration ability of the algorithm to increase, and a smaller $z_{1}$ and a larger $z_{2}$ can cause the search of the algorithm to bias towards the global optimum position and enhance the precision of the solution. This analysis shows that the combined effect of these three factors causes the algorithm to tend away from global exploration to local exploitation, and also balances the search scope of the algorithm.

### 3.5. Selection Mechanisms

In the process of algorithm optimization, establishing how to judge the population state more accurately is always an important direction of research. Making a clear plan according to the population state is the only way to ensure that the algorithm is constantly close to the theoretical optimal solution. Qiang Zhang used cosine similarity to dynamically update the mutation operation of DE [52], Moutaz Alazab used CS to convert continuous problems into binary problems [53], and Wei Li et al. used CS to upgrade algorithm speed [54]. In this paper, cosine similarity is introduced to determine the distance of an individual from the current optimal position, the number of individual similarity angles greater than 90 are recorded separately, and the choice of mechanism is made based on the number of recorded CN. When the angle is greater than 90 the cosine similarity is greater than 0, and vice versa. The specific selection mechanism is as follows:

(7) $$\left\{\begin{array}{c} RCI\;\;\;\;if\;\;CN<N/2\\ QI\;\;\;\;\;\;\;\;\;\;\;otherwise\;\; \end{array}\right.$$

where if CN<N/2 indicates that the similarity angle between most of the individuals within the current population and the current optimal position is less than 90 degrees. This reflects the fact that most of the individuals have high degrees of similarity with each other, and there is low population diversity. There also exists the probability of falling into the local optimum. This occurs when the RCI is used to improve the learning degree of the worst position and enhance the diversity of the population.

Meanwhile, if the number of individuals within the population with an angle greater than ninety degrees from the current optimal position is high, then this reflects the fact that the individuals within the population are not similar to the current optimal position that gives better global exploration ability. Under these circumstances, the QI mechanism is used to enhance the local search of the global optimal position, balancing the local and global search ability of the algorithm. This mechanism of selection ensures that each iteration does not completely lose the local exploitation or global exploration capacity, and it dynamically regulates the learning ability of the algorithm.

### 3.6. Flow of the Algorithm

To improve the judgment and learning ability of PSO, this paper proposes a stochastic learning PSO algorithm based on cosine similarity. Firstly, the algorithm is dynamically set up, and secondly, the selection of RCI and QI is carried out according to the characteristics of cosine similarity, jointly ensuring the capacity of the algorithm to globally explore and locally develop and improving the accuracy of the solution. Using the minimization problem as a case study, the specific pseudo-code of the Algorithm 3 is as follows.
**Algorithm 3.** RPSO***Input:****N: population size**T: Matrix of population information**Maxfes: Maximum number of iterations**Dim: Dimension of the problem**Lb: Lower bound of the problem**Ub: Upper bound of the problem**f: objective function****Output:******x_gbest_**: Matrix of newly generated stock information**Fmin: optimal solution**1.*      *The position matrix x is obtained by random initialization based on the set parameters.**2.*      *Calculate the objective function value based on f to obtain the optimal position **x_gbest_**, the optimal solution f_min_**3.*      
*Fes = N;*
*4.*      
*T = 1;*
*5.*      
***While (t <= T)&&(Fes <= Maxfes)***
*6.*          
*Calculate the new population position x according to Equations (1) and (7)*
*7.*      
*Calculate the objective function value according to f to get the current optimal position **x_l_** its subscripts q, update the optimal position **x_gbest_**, the optimal solution f_min_*
*8.*      
*Fes = Fes + N;*
*9.*          
*Nk = 0*
*10.*        
***For i = 1:N***
*11.*      
***If i ≠ q***
*12.*      
*The cosine similarity between the individual and xl was calculated to obtain nc according to Equation (2)*
*13.*      
***End***
*14.*      
***If** nc < 0*
*15.*      
*Nk = Nk + 1;*
*16.*      
*end*
*17.*      
***end***
*18.*        
***If** Nk > N/2*
*19.*      
*Perform according to Algorithm 2.*
*20.*      
*Calculate the objective function value based on f to obtain the optimal position **x_gbest_**, the optimal solution f_min_*
*21.*      
*Fes = Fes + TS;*
*22.*      
***else***
*23.*      
*Perform according to Algorithm 1.*
*24.*      
*Calculate the objective function value based on f to obtain the optimal position **x_gbest_**, the optimal solution f_min_*
*25.*      
*Fes = Fes + N;*
*26.*        
***End***
*27.*      
*t = t + 1;*
*28.*      
***End***
*Return **x_gbest_**, f_min_*

## 4. Performance Tests

This section validates the proposed algorithm in the CEC 2022 test set, compares it with the variant of PSO and the top algorithm in the CEC competition, and conducts ablation experiments.

### 4.1. Comparison with PSO Variants

**Figure 5:**
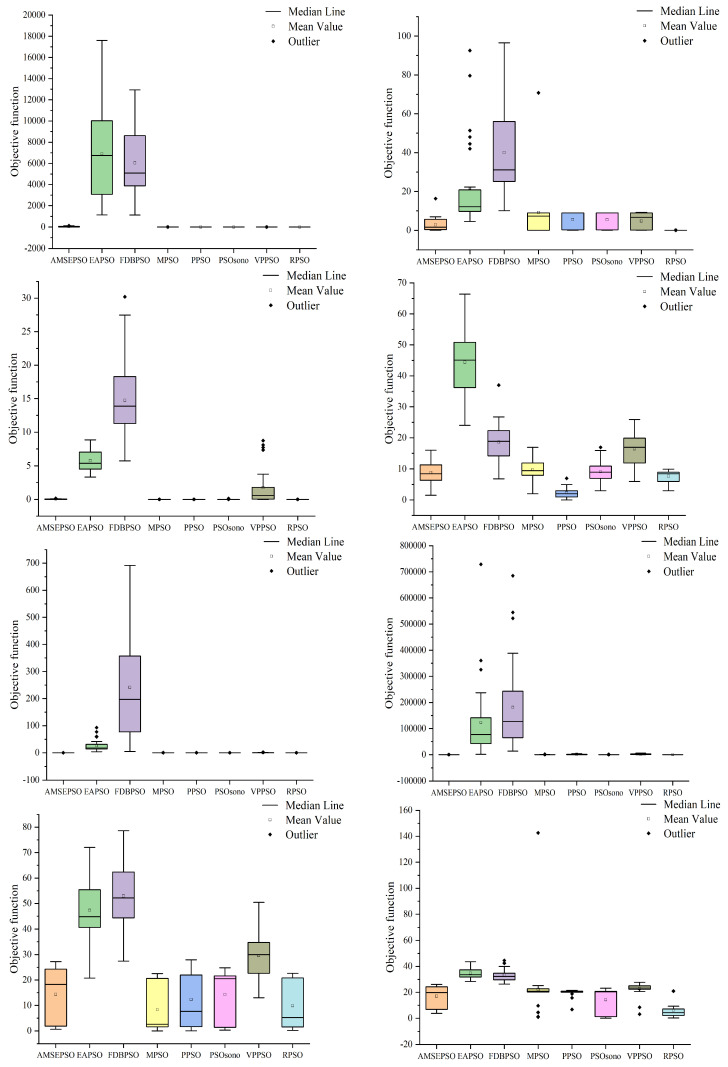

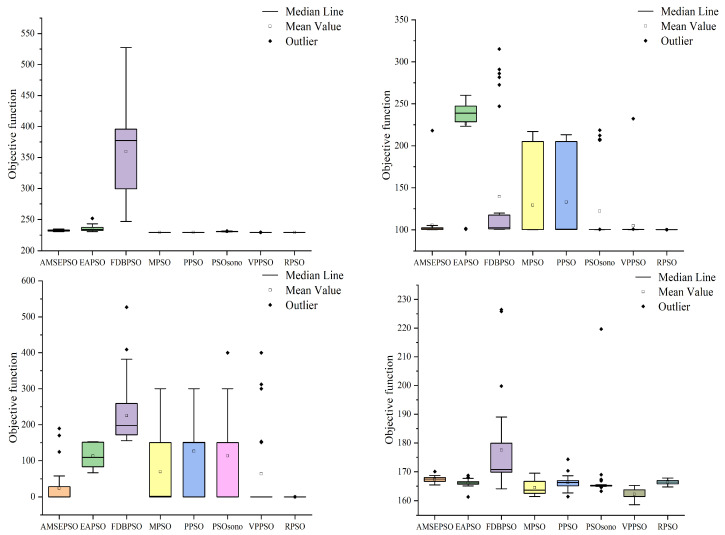
Distribution of solutions for each algorithm in 10 dimensions.

**Figure 6:**
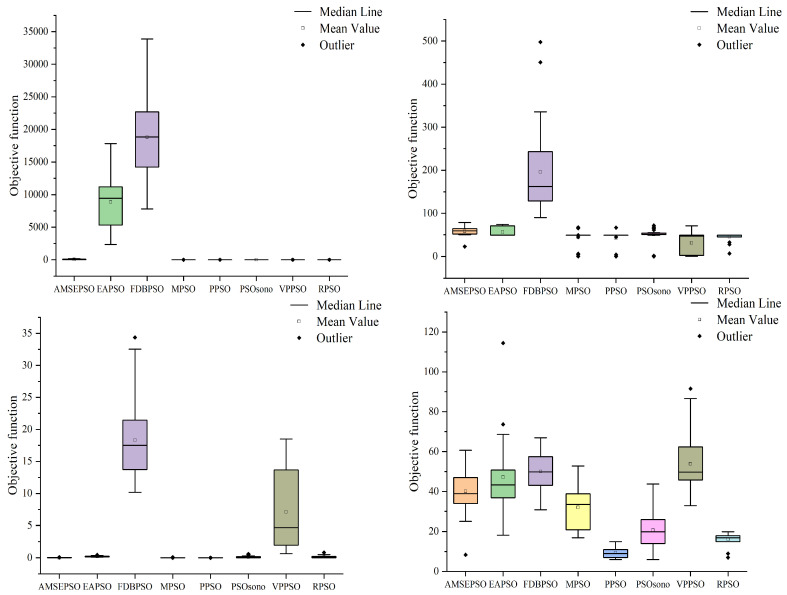

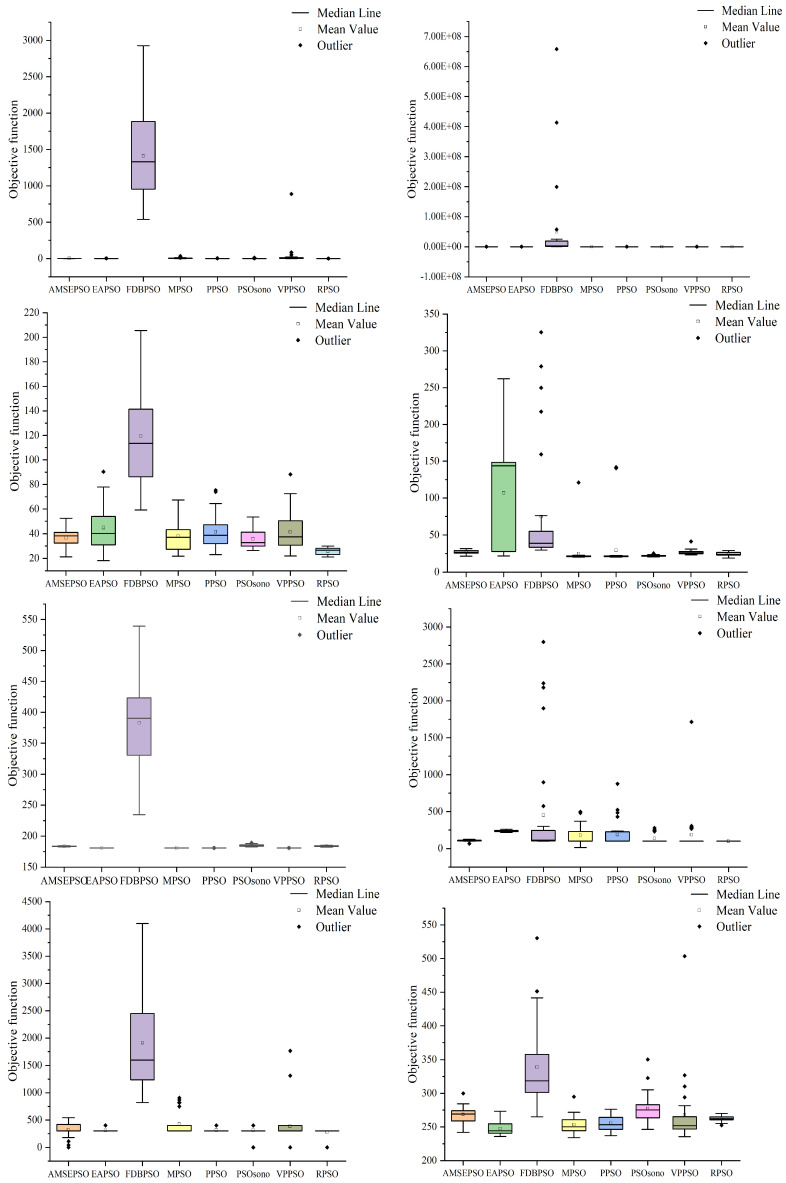
Distribution of solutions for each algorithm in 20 dimensions.

To demonstrate the optimized performance of RPSO, a comparison is conducted with recent variants of PSO algorithms to highlight the novelty and feasibility of RPSO. The compared variants include Adaptive multi-strategy ensemble particle swarm optimization (AMEPSO) [55], Elite archives-driven particle swarm optimization (EAPSO) [56], Improved Phasor Particle Swarm Optimization with Fitness Distance Balance (FDBPSO) [57], Velocity pausing particle swarm optimization (VPPSO) [58], a PSO variant for single-objective numerical optimization (PSOsono) [59], modified particle swarm optimization (MPSO), and Pyramid particle swarm optimization (PPSO) [60]. The internal parameter settings for each algorithm are based on the literature. The population size is set to 100, and the algorithms are tested on 10 and 20 dimensions using a maximum number of evaluations of 200,000 and 1,000,000. The experiments are performed utilizing MATLAB 2019a on an 11th Gen Intel(R) Core(TM) i5-11500 @ 2.70GHz processor. It is worth noting that in the program, all functions of CEC 2022 are minimization functions. We subtract the theoretical optimal value from the optimized result, so the theoretical optimal value becomes 0. The closer the optimization results of each algorithm are to 0, the better the optimization performance. Each algorithm is independently run 30 times, and the optimal value, worst value, median, mean, and standard deviation are recorded. These metrics are used to measure the stability and optimization capability of the algorithm. In simple terms, the smaller the value, the better the optimization effect of the algorithm. The Wilcoxon rank-sum test is used to analyze the differences between RPSO and other algorithms, with a significance level of 0.05. The symbol “+” indicates that RPSO performs better than a certain algorithm in a specific function, “−” indicates the opposite, and “=” shows that the optimization efficiency of the two algorithms is equal. Additionally, the Friedman test is used to measure the overall differences among algorithms and provide a final ranking based on the entire CEC 2022 test set [61]. The detailed optimization results are presented in Table 1 and Table 2. To visualize the distribution of optimization performance for each algorithm, Figure 5 and Figure 6 display the distribution of optimal solutions for each algorithm across different functions.

As shown in Table 1 and Figure 4, in the case of 10 dimensions the RPSO algorithm performs better than the other variants of the PSO algorithm in most cases. Specifically, the optimization standard deviation of the RPSO algorithm is the lowest value in the functions F1(X), F2(X), F5(X), F6(X), F7(X), F10(X), and F12(X). The theoretical optimum is found every time in F1(X), F5(X), and F11(X). In other cases, from small to large, the variance ranks second or third, indicating strong search stability.

The best and worst values of the PSO algorithm variant are contrasted with the best and worst values of the RPSO algorithm, and the number of times that the best and worst values of the PSO algorithm variant are greater than those of the RPSO algorithm in 12 functions is counted. The RPSO algorithm beats the EAPSO algorithm and the FDBPSO algorithm 11 times, the VPPSO algorithm 10 times, the AMSEPSO algorithm 7 times, the MSPO algorithm 5 times, the PPSO and PSOsono algorithms 4 times regarding the optimal value. Regarding the worst value, the RPSO algorithm beats the AMSEPSO, EAPSO, and PDBPSO algorithms 12 times, beats the PSOsono algorithms 11 times, beats the VPPSO algorithm 10 times, and beats the MPSO and PPSO algorithms 9 times and 8 times, respectively. The above information reveals that the RPSO algorithm outperforms the AMSEPSO, EAPSO, FDBPSO, and VPPSO algorithms multiple times in terms of both the optimal value and the worst value. The RPSO algorithm is obviously superior to the above four algorithms. Although the advantages of the MPSO, PPSO and PSOsono algorithms are not obvious in the optimal value, they show great advantages in the worst value. Therefore, the superiority of the proposed algorithm over the variants of the PSO algorithm is verified.

As shown in Table 2 and Figure 5, in the case of 20 dimensions, the RPSO algorithm performs better than other variants of the PSO algorithm in most cases. Specifically, the optimization standard deviation of the RPSO algorithm is the lowest value in functions F1(X), F2(X), F6(X), F10(X), F11(X), and F12(X). The theoretical optimum is found every time in F1(X) and F5(X). In other cases, from small to large, the variance ranks second or third, indicating strong search stability.

The best and worst values of the PSO algorithm variant are compared with the best and worst values of the RPSO algorithm, and the number of times that the best and worst values of the PSO algorithm variant is greater than those of the RPSO algorithm in 12 functions is counted. The RPSO algorithm beats the FDBPSO algorithm 12 times, the EAPSO algorithm 9 times, the VPPSO and AMSEPSO algorithms 8 times, the PPSO algorithm 5 times, and the MPSO and PSOsono algorithms 4 times regarding the optimal value. Regarding the worst value, the RPSO algorithm beats the FDBPSO algorithm 12 times, the EAPSO and VPPSO algorithms 11 times, the AMSEPSO and PPSO algorithms 10 times, and the MSPO and PSOsono algorithms 9 times and 8 times, respectively. The above information shows that the RPSO algorithm beats the AMSEPSO, EAPSO, FDBPSO and VPPSO algorithms many times in terms of the optimal value and the worst value. The RPSO algorithm is clearly superior to the four algorithms mentioned above. While the advantages of the MPSO, PPSO, and PSOsono algorithms may not be obvious in terms of optimal value, they demonstrate significant advantages in terms of worst value. Thus, the proposed algorithm’s superiority over the variants of the PSO algorithm is confirmed. The comparison values mentioned above are similar to the comparison values between the RPSO algorithm and the variants of the PSO algorithm in the case of ten dimensions, indicating the strong robustness of the RPSO algorithm.

### 4.2. Convergence Analysis

**Figure 7:**
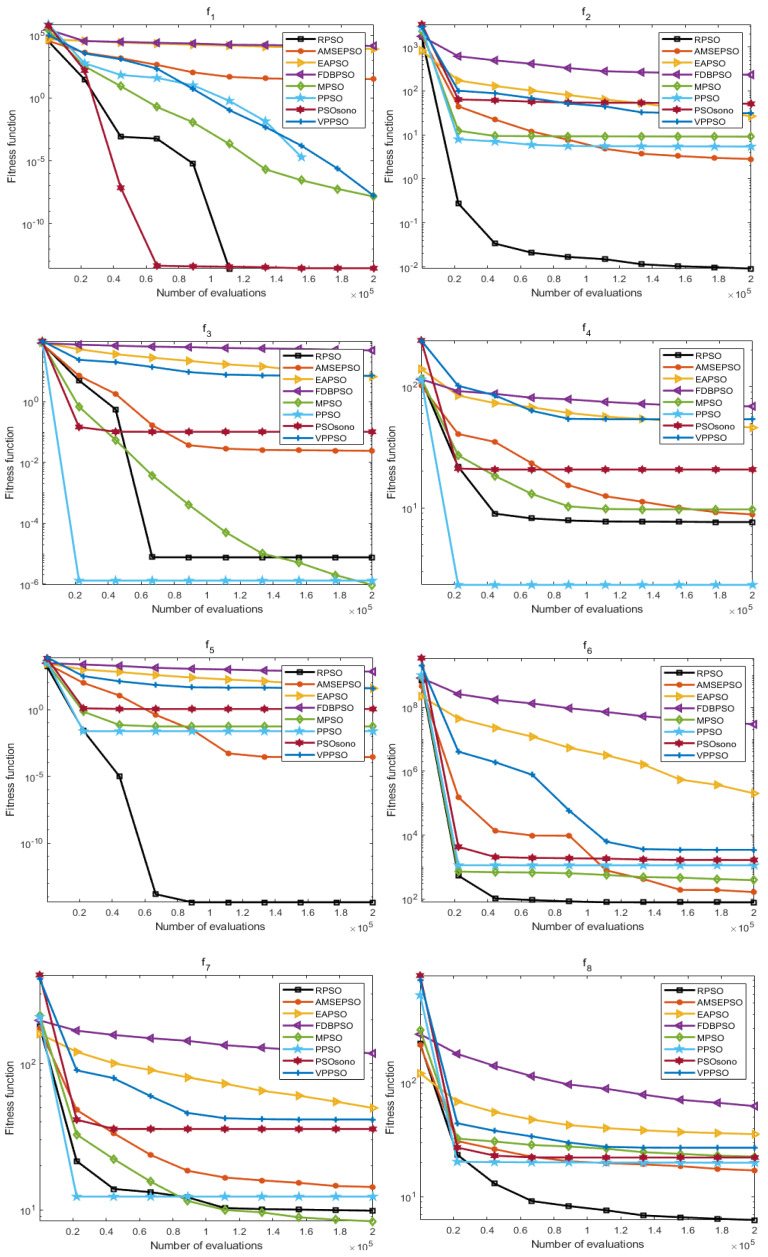

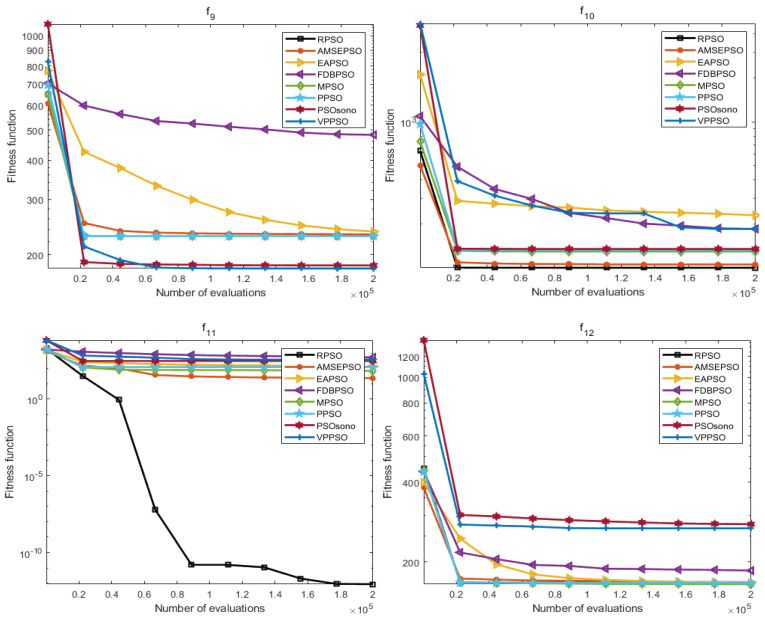
Plot of average convergence effect of each algorithm (dim = 10).

**Figure 8:**
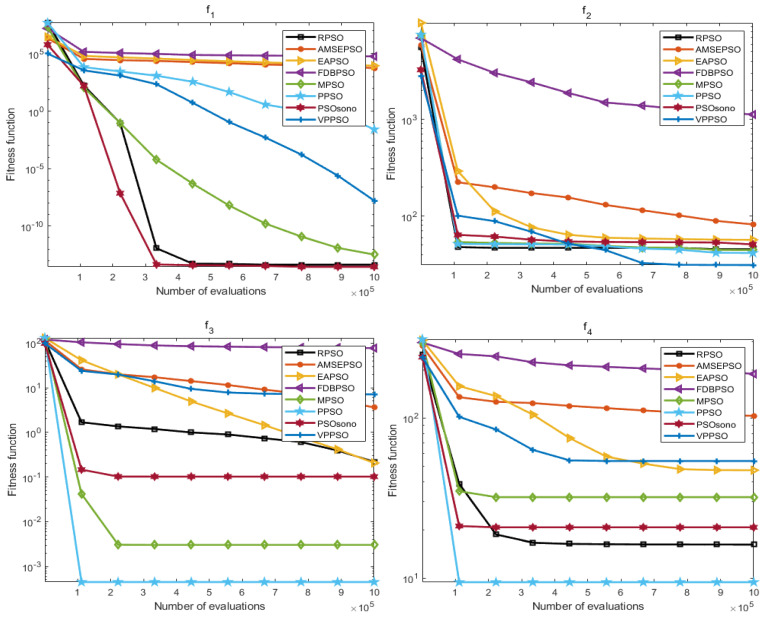

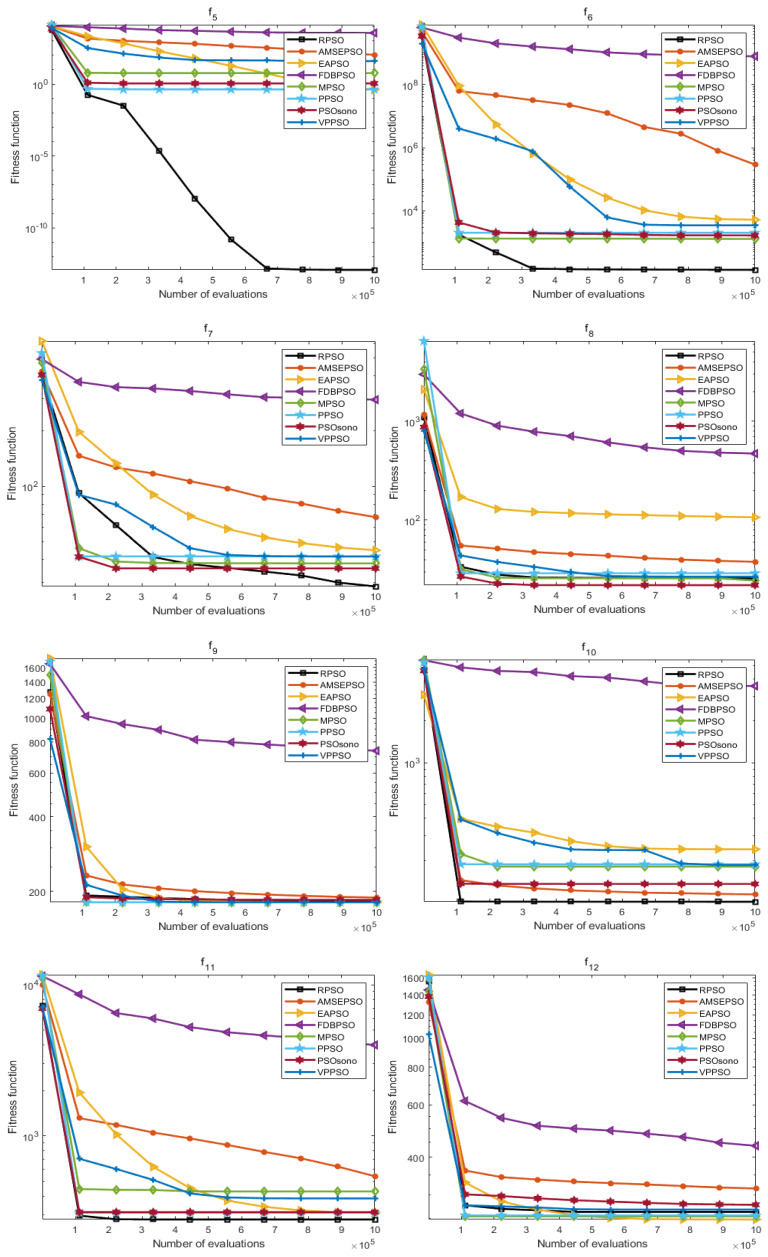
Plot of average convergence effect of each algorithm (dim = 20).

The convergence effect is also an important assessment index for measuring the optimization ability of the algorithm, and the results of the iterative convergence can show the responsiveness of the algorithm in different optimization-seeking environments, which further reflects the learning and adaptability of the algorithm. In this section, the convergence process for the above 30 times is averaged to obtain values, and the average convergence of each algorithm in each function is shown in Figure 7 and Figure 8.

As presented in Figure 7 and Figure 8, the convergence effect of RPSO in both dimensions has some advantages; furthermore, in ten dimensions, the convergence effect of RPSO on F(1–2), F(5–6), F8, and F(10–11) displays a reliable ability to seek optima compared with other algorithms, especially F(2), F(5–6), F(8), and F(11), where the convergence speed of RPSO is more significant. In 20 dimensions, the convergence of RPSO is better in the functions F(1), F(5–7), and F(9–11), particularly in the functions F(5–7), F(10–11), where the convergence speeds of RPSO display a significant advantage. The optimization search effect of RPSO still demonstrates better convergence performance in different dimensions, which verifies the learning and adaptability of RPSO.

### 4.3. Comparison with the Top Algorithm

This subsection aims to reveal the competitive edge of RPSO by comparing it with top algorithms from different years of the CEC competition, including the gaining-sharing knowledge-based algorithm with adaptive parameters hybrid with IMODE (APGSK-IMODE) [62], Eigen crossover in cooperative model of evolutionary algorithms (EA4eig) [63], Improved multi-operator differential evolution (IMODE) [64], Population’s variance-based adaptive differential evolution (PVADE) [65], and the adaptive gaining-sharing knowledge-based algorithm (AGSK) [66]. EA4eig was the champion algorithm in CEC 2022, APGSK-IMODE ranked fourth in CEC 2021, IMODE was the champion in CEC 2020, AGSK was the runner-up in CEC 2020, and PVADE was a recognized algorithm in the competition. The experimental parameters for each algorithm are set consistently as mentioned earlier, and the optimization results are presented in Table 3 and Table 4.

Analyzing Table 3, when the RPSO algorithm was compared with the five most advanced algorithms in ten dimensions, it can be observed that RPSO does not always achieve the best results across the 12 functions when compared with these algorithms. However, the optimization standard deviation of RPSO is the lowest in F(2), F(5), F(9), F(10), and F(11), indicating a strong search stability. Moreover, RPSO outperforms the EA4eig algorithm on the F(10) function, the IMODE algorithm on the F(4) function, the PVADE algorithm on the F(8) and F(10) functions, and the AGSK algorithm on the F(4) and F(10) functions in terms of the optimal value. RPSO also outperforms the APGSK-IMODE algorithm on the F(10) function; the EA4eig algorithm on the F(2) function; the IMODE algorithm on the F(4), F(5), F(10), and F(11) functions; and the PVADE algorithm on the F(2), F(8), and F(11) functions. Additionally, RPSO outperforms the AGSK algorithm on the F(2), F(4), F(5), and F(11) functions.

Examining Table 4, when comparing the RPSO algorithm with the top algorithms in 20 dimensions, it can be observed that RPSO does not always achieve the best results across the 12 functions when compared with these algorithms. However, RPSO exhibits better performance compared to the 10-dimensional case. The optimization standard deviation is the lowest in F(1), F(5), and F(7), indicating strong search stability. Additionally, RPSO outperforms the APGSK-IMODE algorithm on the F(1), F(5), and F(11) functions; the EA4eig algorithm on the F(11) function; and the IMODE algorithm on the F(3), F(4), F(5), F(7), F(8), F(10), and F(11) functions in terms of the optimal value. RPSO also outperforms the AGSK algorithm on the F(4), F(5), and F(11) functions. In terms of the worst value, RPSO outperforms the APGSK-IMODE algorithm on the F(1) and F(4) functions; the EA4eig algorithm on the F(10) and F(11) functions; and the IMODE algorithm on the F(4), F(5), F(7), F(10), and F(11) functions. RPSO also outperforms the PVADE algorithm on the F(9) and F(10) functions; and the AGSK algorithm on the F(1), F(4), F(5), and F(11) functions.

### 4.4. Ablation Experiments

In order to further confirm the effectiveness of strategy fusion, this subsection conducts ablation experiments one by one on the mechanisms proposed in this paper. The PSO that only incorporates random exchange learning is set as PSO-1, the PSO algorithm that only incorporates QI is set as PSO-2, and the PSO algorithm that fuses the two mechanisms and does not use the selection mechanism is named PSO-3. It is worth mentioning that the parameter settings of RPSO are derived from previous research by other scholars, and thus are not included in the scope of validation. The other experimental parameters are consistent with those described, and the specific optimization results are shown in Table 5.

As can be seen from the Table 5, except for F12, RPSO has optimal values in all other functions, especially in the functions F1–3, F5, F7–11, where the indexes of RPSO are optimal in these functions. The other variants are all able to find the theoretical optimal values in the functions F1–3, F5, and F11. This verifies that the incorporation of the various mechanisms has a certain degree of rationality. Taken together, RPSO has certain advantages in the arrangement and combination of integrated mechanisms, verifying the feasibility and effectiveness of RPSO.

## 5. Classification Experiments

In this section, we optimize the hyperparameters of a CNN for enhancing the diagnostic rate through RPSO and verify the feasibility of this model using two datasets.

### 5.1. Means

In this study, the optimization of hyperparameters was performed using RPSO. The accuracy and convergence of CNNs are heavily determined by hyperparameters [67]. Properly choosing hyperparameters is crucial, as they are determined by the particular use of the CNN. Some commonly employed hyperparameters for training CNNs include the learning rate, the number of epochs, the momentum, and the regularization factor. The learning rate governs the step size of the gradient descent algorithm, dictating how quickly or slowly the network parameters are updated. An increased learning rate facilitates faster convergence yet may result in overshooting the optimal solution. On the other hand, a decreased learning rate may take more iterations to converge, but it can yield more accurate results. The number of epochs specifies the frequency of updating the network parameters based on the training dataset. Striking a balance between not training enough epochs and overtraining, which could lead to underfitting and overfitting, is crucial. Momentum is a parameter that governs the influence of previous weight updates on the current weight update. It helps to accelerate convergence and overcome local minima. Regularization is a technique employed to prevent overfitting in the network. It accomplishes this by adding a regularization term to the loss function, penalizing complex models and promoting simpler models that can be generalized to unseen data more effectively. Optimizing hyperparameters is imperative to achieve the optimal performance of the CNN. Techniques such as RPSO can be utilized to explore the hyperparameter space and identify the most suitable values for the specific problem at hand.

### 5.2. Training

In this section, two datasets, CT (https://github.com/UCSD-AI4H/COVID-CT, accessed on 20 March 2023) and XY (https://www.kaggle.com/datasets/paultimothymooney/chest-xray-pneumonia, accessed on 20 March 2023), were selected to validate the algorithm’s ability to optimize the CNN. Overall, 70% of the data were utilized for training, while the remaining 30% were allocated for network testing. The proposed architecture aims to distinguish between normal and abnormal images. The dataset was randomly divided into training and testing sets. Through data augmentation techniques, all images were resized to 224 × 224 × 3 and converted into color images. A six convolutional layer structure was employed for image classification, with Stochastic Gradient Descent (SGD) training used to adjust parameters and RPSO for hyperparameter optimization during the training process.

### 5.3. Testing

Firstly, the proposed network was augmented with data and trained by resizing the image to 224 × 224 × 3. Then, the test image was fed into the trained CNN that had already optimized all the parameters of Convolutional Layers (CLs) and Fully Connected Layers (FCL). The CNN starts by extracting image characteristics and then categorizes them into appropriate groups through FCL and soft-max classifiers.

### 5.4. Experiments

**Figure 9:**
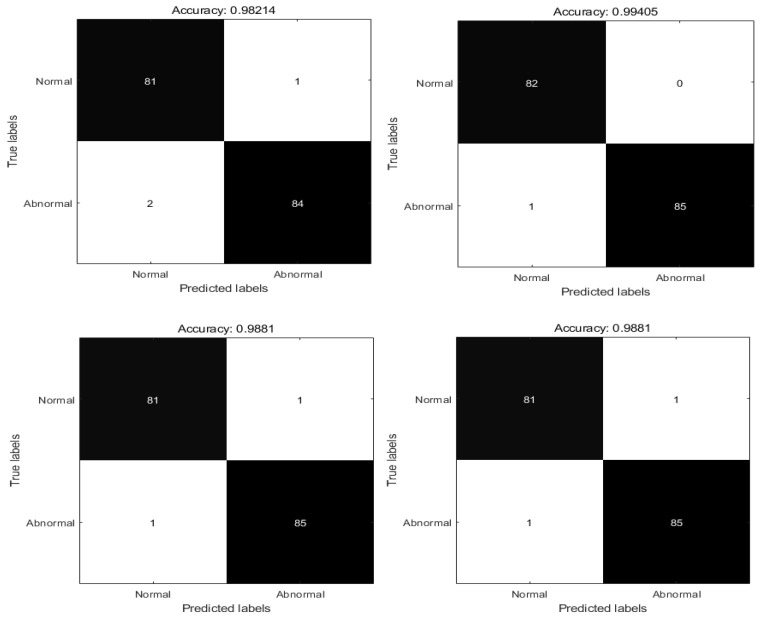

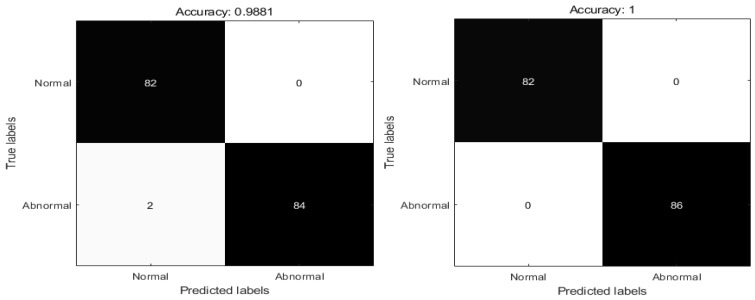
Confusion matrix for each algorithm with the XY dataset.

**Figure 10:**
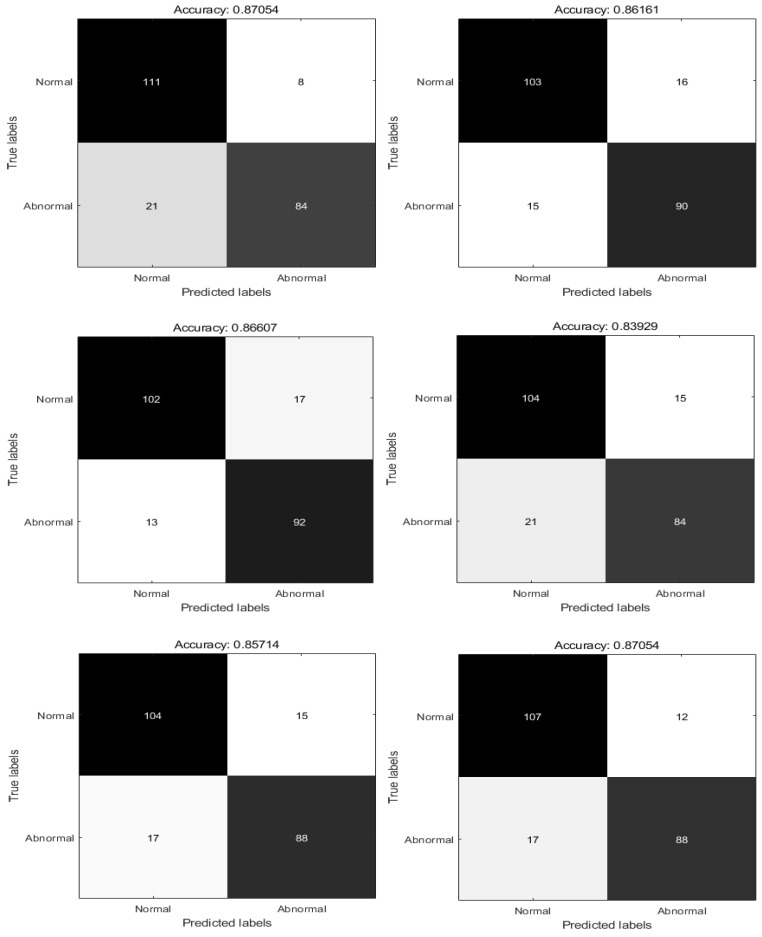
Confusion matrix for each algorithm with the XY dataset.

To show the diagnostic performance of RPSO, the diagnostic effectiveness of RPSO was compared with that of PSO, the whale optimization algorithm (WOA) [68], the grey wolf optimizer (GWO), the hybrid differential evolution algorithm and particle swarm optimization (DEPSO) [69], and the advanced squirrel search optimization algorithm (ASSOA) [70]. Each algorithm was run independently for ten epochs; the best value, mean value, and worst value of every algorithm were recorded; and the confusion matrix of the optimal value, Accuracy, Precision, Recall, and F1 Score [71] was produced. The specific results are shown in Table 6 and Figure 9 and Figure 10.

According to Table 6 and Figure 9 and Figure 10, in the XY data set the algorithm accuracy is sorted from low to high. The results show that the accuracy rate of the PSO algorithm in the diagnosis of pneumonia is 98.21%, the number of normal correct diagnoses times is 85, and the number of correct diagnoses of COVID-19 is 84. The accuracy rate of the WOA algorithm in the diagnosis of pneumonia is 98.81%, the number of normal and correct diagnoses times is 81, and the number of times with correct diagnosis of COVID-19 is 85. The accuracy of the DEPSO algorithm in the diagnosis of pneumonia is 98.81%, the number of normal correct diagnoses is 81, and the number of correct diagnoses of COVID-19 is 85. The accuracy of the ASSOS algorithm in the diagnosis of pneumonia is 98.81%, the number of normal correct diagnoses is 82, and the number of correct diagnoses of COVID-19 is 84. The accuracy of the GWO algorithm in the diagnosis of pneumonia is 99.40%, and the number of normal correct diagnoses is 82, while the number of times with the correct diagnosis of COVID-19 is 85. The accuracy rate of the RPSO algorithm in diagnosing pneumonia is 100%. The number of correct diagnoses for normal cases is 82, while the number of correct diagnoses for COVID-19 cases is 86.

In the CT dataset, the algorithm accuracy is ranked from low to high. The results indicate that the ASSOA algorithm has an accuracy of 85.71% in diagnosing pneumonia, the number of normal correct diagnoses is 104, and the number of correct diagnoses of COVID-19 cases is 88. The DEPSO algorithm has an accuracy rate of 83.92% in diagnosing pneumonia. The number of correct diagnoses for normal cases is 104, and the number of correct diagnoses for COVID-19 cases is 84. The GWO algorithm has an accuracy of 86.16% in diagnosing pneumonia. The number of correct diagnoses for normal cases is 103, and the number of correct diagnoses for COVID-19 cases is 90. The WOA algorithm has an accuracy of 86.61% in diagnosing pneumonia. The number of correct diagnoses for normal cases is 102, and the number of correct diagnoses for COVID-19 cases is 92. The accuracy of the PSO algorithm in diagnosing pneumonia is 87.05%. The number of correct diagnoses for normal cases is 111, and for COVID-19 cases it is 84. The accuracy rate of the RPSO algorithm in diagnosing pneumonia is also 87.05%. The number of correct diagnoses for normal cases is 107, and for COVID-19 cases it is 88.

It can be seen that, compared with other algorithms, the RPSO algorithm has the highest accuracy in both data sets, surpassing all of the compared algorithms. Although the accuracy of the PSO algorithm in the CT data set is equal to that of RPSO, with both ranking first, it has the lowest accuracy in the XY data set and is highly unstable. It is concluded that RPSO has obvious superiority and good robustness.

## 6. Conclusions

To further enhance the global optimization ability of the particle swarm algorithm and its applicability in threshold segmentation problems, this paper proposes an RPSO based on cosine similarity. This algorithm introduces the RCI mechanism to enhance the mechanism and the global search capability of the algorithm. Utilizing cosine similarity for RCI and QI selection, the population information is effectively and dynamically updated, thereby improving the global and local optimization capabilities of the algorithm. RPSO is evaluated based on the CEC 2022 test set and compared with other PSO variants and top algorithms in the CEC community. In comparison with other variants of PSO, RPSO is ranked top at 7.9167 and 2.5833 in 10 and 20 dimensions, respectively. When compared with the top algorithms, RPSO achieves optimal values in F(1), F(5), and F(11), performing on par with the leading algorithms. The results confirm that RPSO exhibits strong competitive and global optimization capabilities. In the optimization of CNN classification experiments, RPSO demonstrates the highest classification accuracy of 100% on the XY dataset, with an average accuracy exceeding 98%. On the CT dataset, the highest accuracy reached is over 87%, with an average accuracy of above 85%. RPSO demonstrates excellent diagnostic performance based on two disease datasets compared to other basic algorithms and algorithm variants, highlighting its practical value.

Despite the promising optimization results achieved by RPSO based on the CEC 2022 test set, some limitations still exist. For instance, in functions F(3) and F(4), RPSO exhibits some shortcomings in search accuracy, indicating the need to improve its local search capability while maintaining its global search ability, as RPSO is not capable of making intelligent choices based on the optimization-seeking environment. Additionally, optimizing the hyperparameters of the CNN to significantly enhance the classification accuracy is a challenging task, as optimization is a major problem that takes an inordinate amount of time to ensure. Therefore, the next step of this research focuses on three main areas:

- Designing smarter judgment mechanisms for rational global and local searching.
- Incorporating efficient parameter estimation schemes to reduce the time consumption of the optimization process.
- Optimizing the architecture of the neural network to enable automatic architectural tuning for different datasets.
- Applying the algorithm to practical scenarios in high demand today, such as 6G base station deployment, optimization of new energy systems, and structural design optimization.

## Figures and Tables

**Table 1:** Ten dimensions with PSO algorithm variants.

F	Index	AMSE PSO	EAPSO	FDBPSO	MPSO	PPSO	PSOsono	VPPSO	RPSO
F1(x)	Best	0.00 × 10^00^	1.13 × 10^03^	1.12 × 10^03^	0.00 × 10^00^	0.00 × 10^00^	0.00 × 10^00^	1.93 × 10^−9^	**0.00 × 10^00^**
	Worst	1.26 × 10^02^	1.76 × 10^04^	1.29 × 10^04^	2.25 × 10^−07^	0.00 × 10^00^	0.00 × 10^00^	7.62 × 10^−9^	**0.00 × 10^00^**
	Median	2.49 × 10^01^	6.75 × 10^03^	5.09 × 10^03^	1.38 × 10^−10^	0.00 × 10^00^	0.00 × 10^00^	4.34 × 10^−9^	**0.00 × 10^00^**
	Mean	3.25 × 10^01^	6.90 × 10^03^	6.04 × 10^03^	1.40 × 10^−8^	0.00 × 10^00^	0.00 × 10^00^	4.39 × 10^−9^	**0.00 × 10^00^**
	Standard	3.56 × 10^01^	4.25 × 10^03^	2.94 × 10^03^	4.57 × 10^−8^	0.00 × 10^00^	0.00 × 10^00^	1.28 × 10^−9^	**0.00 × 10^00^**
	Contest	1.94 × 10^−9^ (+)	1.21 × 10^−12^ (+)	1.21 × 10^−12^ (+)	1.21 × 10^−12^ (+)	N/A (=)	N/A (=)	1.21 × 10^−12^ (+)	
F2(x)	Best	9.47 × 10^−03^	4.54 × 10^00^	1.01 × 10^01^	8.20 × 10^−8^	2.45 × 10^−04^	0.00 × 10^00^	1.45 × 10^−02^	1.71 × 10^−07^
	Worst	1.63 × 10^01^	9.26 × 10^01^	9.65 × 10^01^	7.08 × 10^01^	8.92 × 10^00^	1.28 × 10^01^	9.28 × 10^00^	**7.32 × 10^−02^**
	Median	1.62 × 10^00^	1.21 × 10^01^	3.11 × 10^01^	7.32 × 10^00^	8.92 × 10^00^	3.99 × 10^00^	6.70 × 10^00^	**5.91 × 10^−04^**
	Mean	2.83 × 10^00^	2.17 × 10^01^	4.00 × 10^01^	9.12 × 10^00^	5.44 × 10^00^	4.65 × 10^00^	4.81 × 10^00^	**9.12 × 10^−03^**
	Standard	3.58 × 10^00^	2.16 × 10^01^	2.28 × 10^01^	1.73 × 10^01^	4.06 × 10^00^	3.69 × 10^00^	4.33 × 10^00^	**1.80 × 10^−02^**
	Contest	9.92 × 10^−11^ (+)	3.02 × 10^−11^ (+)	3.02 × 10^−11^ (+)	3.59 × 10^−07^ (+)	7.41 × 10^−10^ (+)	7.13 × 10^−05^ (+)	2.15 × 10^−10^ (+)	
F3(x)	Best	0.00 × 10^00^	3.31 × 10^00^	5.74 × 10^00^	2.09 × 10^−11^	0.00 × 10^00^	0.00 × 10^00^	1.44 × 10^−04^	**0.00 × 10^00^**
	Worst	1.45 × 10^−11^	8.85 × 10^00^	3.02 × 10^01^	2.33 × 10^−05^	1.57 × 10^−05^	1.22 × 10^−11^	8.73 × 10^00^	8.79 × 10^−05^
	Median	5.53 × 10^−03^	5.38 × 10^00^	1.39 × 10^01^	3.06 × 10^−8^	0.00 × 10^00^	2.56 × 10^−06^	5.67 × 10^−11^	1.42 × 10^−06^
	Mean	2.40 × 10^−02^	5.75 × 10^00^	1.47 × 10^01^	8.73 × 10^−07^	1.30 × 10^−06^	5.53 × 10^−03^	1.74 × 10^00^	7.62 × 10^−06^
	Standard	3.41 × 10^−02^	1.55 × 10^00^	5.78 × 10^00^	4.25 × 10^−06^	3.55 × 10^−06^	2.24 × 10^−02^	2.65 × 10^00^	1.90 × 10^−05^
	Contest	6.48 × 10^−11^ (=)	2.95 × 10^−11^ (+)	2.95 × 10^−11^ (+)	9.43 × 10^−03^ (−)	6.38 × 10^−05^ (−)	4.67 × 10^−11^ (=)	2.95 × 10^−11^ (+)	
F4(X)	Best	1.55 × 10^00^	2.40 × 10^01^	6.81 × 10^00^	1.99 × 10^00^	0.00 × 10^00^	2.98 × 10^00^	5.97 × 10^00^	2.98 × 10^00^
	Worst	1.60 × 10^01^	6.64 × 10^01^	3.70 × 10^01^	1.69 × 10^01^	6.96 × 10^00^	1.69 × 10^01^	2.59 × 10^01^	9.95 × 10^00^
	Median	8.46 × 10^00^	4.51 × 10^01^	1.88 × 10^01^	9.45 × 10^00^	1.99 × 10^00^	8.95 × 10^00^	1.69 × 10^01^	8.46 × 10^00^
	Mean	8.82 × 10^00^	4.44 × 10^01^	1.86 × 10^01^	9.72 × 10^00^	2.32 × 10^00^	9.09 × 10^00^	1.64 × 10^01^	7.63 × 10^00^
	Standard	3.45 × 10^00^	1.03 × 10^01^	6.25 × 10^00^	3.82 × 10^00^	1.60 × 10^00^	3.07 × 10^00^	5.12 × 10^00^	2.08 × 10^00^
	Contest	1.58 × 10^−11^ (=)	2.93 × 10^−11^ (+)	3.72 × 10^−10^ (+)	1.94 × 10^−03^ (+)	4.00 × 10^−10^ (−)	1.64 × 10^−11^ (=)	5.96 × 10^−10^ (+)	
F5(X)	Best	0.00 × 10^00^	3.75 × 10^00^	4.85 × 10^00^	0.00 × 10^00^	0.00 × 10^00^	0.00 × 10^00^	8.43 × 10^−10^	**0.00 × 10^00^**
	Worst	2.69 × 10^−03^	9.28 × 10^01^	6.91 × 10^02^	4.54 × 10^−11^	4.54 × 10^−11^	8.95 × 10^−02^	2.82 × 10^00^	**0.00 × 10^00^**
	Median	0.00 × 10^00^	1.77 × 10^01^	1.97 × 10^02^	0.00 × 10^00^	0.00 × 10^00^	0.00 × 10^00^	1.53 × 10^−9^	**0.00 × 10^00^**
	Mean	2.77 × 10^−04^	2.58 × 10^01^	2.42 × 10^02^	5.44 × 10^−02^	2.41 × 10^−02^	2.98 × 10^−03^	3.99 × 10^−11^	**0.00 × 10^00^**
	Standard	7.17 × 10^−04^	2.10 × 10^01^	1.77 × 10^02^	1.38 × 10^−11^	8.57 × 10^−02^	1.63 × 10^−02^	6.90 × 10^−11^	**0.00 × 10^00^**
	Contest	2.16 × 10^−04^ (+)	1.72 × 10^−12^ (+)	1.72 × 10^−12^ (+)	1.26 × 10^−9^ (+)	1.31 × 10^−03^ (+)	1.00 × 10^00^ (=)	1.72 × 10^−12^ (+)	
F6(X)	Best	5.43 × 10^01^	1.84 × 10^03^	1.39 × 10^04^	2.46 × 10^01^	9.30 × 10^00^	4.72 × 10^00^	1.22 × 10^02^	**2.70 × 10^00^**
	Worst	4.98 × 10^02^	7.29 × 10^−14^	6.85 × 10^−14^	1.96 × 10^03^	4.18 × 10^03^	1.37 × 10^03^	6.25 × 10^03^	**1.96 × 10^02^**
	Median	1.26 × 10^02^	7.69 × 10^04^	1.27 × 10^−14^	1.57 × 10^02^	5.23 × 10^02^	5.01 × 10^01^	1.82 × 10^03^	**6.32 × 10^01^**
	Mean	1.64 × 10^02^	1.24 × 10^−14^	1.81 × 10^−14^	3.70 × 10^02^	1.13 × 10^03^	1.47 × 10^02^	2.38 × 10^03^	**7.83 × 10^01^**
	Standard	1.11 × 10^02^	1.44 × 10^−14^	1.67 × 10^05^	5.25 × 10^02^	1.36 × 10^03^	2.79 × 10^02^	1.92 × 10^03^	**6.02 × 10^01^**
	Contest	3.01 × 10^−04^ (+)	3.02 × 10^−11^ (+)	3.02 × 10^−11^ (+)	4.23 × 10^−03^ (+)	5.61 × 10^−05^ (+)	6.10 × 10^−11^ (=)	2.37 × 10^−10^ (+)	
F7(X)	Best	6.35 × 10^−11^	2.07 × 10^01^	2.74 × 10^01^	6.44 × 10^−05^	2.28 × 10^−02^	2.81 × 10^−11^	1.30 × 10^01^	1.96 × 10^−11^
	Worst	2.73 × 10^01^	7.21 × 10^01^	7.86 × 10^01^	2.25 × 10^01^	2.79 × 10^01^	2.48 × 10^01^	5.04 × 10^01^	2.26 × 10^01^
	Median	1.83 × 10^01^	4.48 × 10^01^	5.22 × 10^01^	2.62 × 10^00^	7.69 × 10^00^	2.05 × 10^01^	3.00 × 10^01^	**5.20 × 10^00^**
	Mean	1.43 × 10^01^	4.73 × 10^01^	5.29 × 10^01^	8.33 × 10^00^	1.23 × 10^01^	1.43 × 10^01^	2.96 × 10^01^	9.88 × 10^00^
	Standard	1.10 × 10^01^	1.17 × 10^01^	1.27 × 10^01^	9.35 × 10^00^	1.04 × 10^01^	9.93 × 10^00^	8.52 × 10^00^	9.20 × 10^00^
	Contest	5.94 × 10^−02^ (=)	6.70 × 10^−11^ (+)	3.02 × 10^−11^ (+)	5.69 × 10^−11^ (=)	1.71 × 10^−11^ (=)	2.34 × 10^−11^ (=)	3.50 × 10^−9^ (+)	
F8(X)	Best	3.80 × 10^00^	2.83 × 10^01^	2.63 × 10^01^	9.43 × 10^−11^	6.80 × 10^00^	1.63 × 10^−11^	3.10 × 10^00^	2.21 × 10^−11^
	Worst	2.61 × 10^01^	4.35 × 10^01^	4.45 × 10^01^	1.43 × 10^02^	2.15 × 10^01^	2.32 × 10^01^	2.78 × 10^01^	**2.10 × 10^01^**
	Median	1.98 × 10^01^	3.36 × 10^01^	3.23 × 10^01^	2.09 × 10^01^	2.04 × 10^01^	2.03 × 10^01^	2.33 × 10^01^	**4.36 × 10^00^**
	Mean	1.70 × 10^01^	3.47 × 10^01^	3.33 × 10^01^	2.23 × 10^01^	1.99 × 10^01^	1.43 × 10^01^	2.28 × 10^01^	**6.25 × 10^00^**
	Standard	8.23 × 10^00^	4.10 × 10^00^	4.77 × 10^00^	2.40 × 10^01^	2.69 × 10^00^	9.38 × 10^00^	4.99 × 10^00^	6.40 × 10^00^
	Contest	1.17 × 10^−05^ (+)	3.02 × 10^−11^ (+)	3.02 × 10^−11^ (+)	4.42 × 10^−06^ (+)	2.57 × 10^−07^ (+)	3.64 × 10^−02^ (+)	5.07 × 10^−10^ (+)	
F9(X)	Best	2.31 × 10^02^	2.31 × 10^02^	2.47 × 10^02^	2.29 × 10^02^	2.29 × 10^02^	2.30 × 10^02^	2.29 × 10^02^	**2.29 × 10^02^**
	Worst	2.35 × 10^02^	2.52 × 10^02^	5.27 × 10^02^	2.29 × 10^02^	2.29 × 10^02^	2.31 × 10^02^	2.29 × 10^02^	**2.29 × 10^02^**
	Median	2.32 × 10^02^	2.34 × 10^02^	3.77 × 10^02^	2.29 × 10^02^	2.29 × 10^02^	2.31 × 10^02^	2.29 × 10^02^	**2.29 × 10^02^**
	Mean	2.32 × 10^02^	2.35 × 10^02^	3.60 × 10^02^	2.29 × 10^02^	2.29 × 10^02^	2.31 × 10^02^	2.29 × 10^02^	**2.29 × 10^02^**
	Standard	1.07 × 10^00^	4.26 × 10^00^	6.49 × 10^01^	0.00 × 10^00^	0.00 × 10^00^	3.41 × 10^−11^	1.58 × 10^−06^	**0.00 × 10^00^**
	Contest	1.21 × 10^−12^ (+)	1.21 × 10^−12^ (+)	1.21 × 10^−12^ (+)	N/A (=)	N/A (=)	1.21 × 10^−12^ (+)	1.21 × 10^−12^ (+)	
F10(X)	Best	1.00 × 10^02^	1.01 × 10^02^	1.01 × 10^02^	1.00 × 10^02^	1.00 × 10^02^	1.00 × 10^02^	1.00 × 10^02^	**1.00 × 10^02^**
	Worst	2.18 × 10^02^	2.60 × 10^02^	3.15 × 10^02^	2.17 × 10^02^	2.13 × 10^02^	2.19 × 10^02^	2.32 × 10^02^	**1.00 × 10^02^**
	Median	1.02 × 10^02^	2.39 × 10^02^	1.03 × 10^02^	1.00 × 10^02^	1.01 × 10^02^	1.00 × 10^02^	1.00 × 10^02^	**1.00 × 10^02^**
	Mean	1.06 × 10^02^	2.26 × 10^02^	1.40 × 10^02^	1.29 × 10^02^	1.33 × 10^02^	1.22 × 10^02^	1.05 × 10^02^	**1.00 × 10^02^**
	Standard	2.13 × 10^01^	4.33 × 10^01^	7.33 × 10^01^	4.92 × 10^01^	5.04 × 10^01^	4.46 × 10^01^	2.41 × 10^01^	**5.59 × 10^−02^**
	Contest	5.53 × 10^−8^ (+)	3.02 × 10^−11^ (+)	3.02 × 10^−11^ (+)	1.69 × 10^−9^ (+)	4.98E-1 (+)1	3.26 × 10^−07^ (+)	2.20 × 10^−07^ (+)	**0.00 × 10^00^**
F11(X)	Best	0.00 × 10^00^	6.70 × 10^01^	1.55 × 10^02^	5.78 × 10^−11^	0.00 × 10^00^	0.00 × 10^00^	4.29 × 10^−04^	**0.00 × 10^00^**
	Worst	1.89 × 10^02^	1.52 × 10^02^	5.27 × 10^02^	3.00 × 10^02^	3.00 × 10^02^	4.00 × 10^02^	4.00 × 10^02^	**0.00 × 10^00^**
	Median	0.00 × 10^00^	1.09 × 10^02^	1.98 × 10^02^	1.87 × 10^00^	1.50 × 10^02^	0.00 × 10^00^	5.48 × 10^−04^	**0.00 × 10^00^**
	Mean	2.41 × 10^01^	1.13 × 10^02^	2.26 × 10^02^	6.99 × 10^01^	1.26 × 10^02^	1.13 × 10^02^	6.39 × 10^01^	**0.00 × 10^00^**
	Standard	5.06 × 10^01^	3.00 × 10^01^	8.51 × 10^01^	9.28 × 10^01^	1.05 × 10^02^	1.51 × 10^02^	1.25 × 10^02^	**0.00 × 10^00^**
	Contest	5.70 × 10^−03^ (+)	1.68 × 10^−11^ (+)	1.68 × 10^−11^ (+)	1.68 × 10^−11^ (+)	7.30 × 10^−04^ (+)	3.48 × 10^−11^ (=)	1.68 × 10^−11^ (+)	**0.00 × 10^00^**
F12(X)	Best	1.65 × 10^02^	1.61 × 10^02^	1.64 × 10^02^	1.61 × 10^02^	1.61 × 10^02^	1.63 × 10^02^	1.59 × 10^02^	1.65 × 10^02^
	Worst	1.70 × 10^02^	1.69 × 10^02^	2.26 × 10^02^	1.70 × 10^02^	1.74 × 10^02^	2.20 × 10^02^	1.65 × 10^02^	1.68 × 10^02^
	Median	1.67 × 10^02^	1.66 × 10^02^	1.71 × 10^02^	1.64 × 10^02^	1.66 × 10^02^	1.65 × 10^02^	1.61 × 10^02^	1.66 × 10^02^
	Mean	1.67 × 10^02^	1.66 × 10^02^	1.77 × 10^02^	1.64 × 10^02^	1.66 × 10^02^	1.67 × 10^02^	1.62 × 10^02^	1.66 × 10^02^
	Standard	1.07 × 10^00^	1.27 × 10^00^	1.54 × 10^01^	2.40 × 10^00^	2.38 × 10^00^	9.96 × 10^00^	1.58 × 10^00^	8.15 × 10^−11^
	Contest	4.22 × 10^−04^ (+)	5.49 × 10^−11^ (=)	5.46 × 10^−9^ (+)	1.44 × 10^−03^ (−)	4.12 × 10^−11^ (=)	4.08 × 10^−05^ (+)	4.98 × 10^−11^ (−)	
+/=/−		9/3/0	11/1/0	12/0/0	2008/2/2	2006/4/2	5/7/0	11/0/1	
Rank		3.83	6.83	7.67	3.83	3.83	3.67	4.42	1.92

**Table 2:** Twenty dimensions with PSO algorithm variants.

F	Index	AMSE PSO	EAPSO	FDBPSO	MPSO	PPSO	PSOsono	VPPSO	RPSO
F1(x)	Best	0.00 × 10^00^	2.34 × 10^03^	7.81 × 10^03^	0.00 × 10^00^	0.00 × 10^00^	0.00 × 10^00^	1.02 × 10^−8^	**0.00 × 10^00^**
	Worst	1.87 × 10^02^	1.78 × 10^04^	3.39 × 10^04^	0.00 × 10^00^	7.59 × 10^−11^	0.00 × 10^00^	2.35 × 10^−8^	**0.00 × 10^00^**
	Median	4.17 × 10^01^	9.44 × 10^03^	1.88 × 10^04^	0.00 × 10^00^	0.00 × 10^00^	0.00 × 10^00^	1.59 × 10^−8^	**0.00 × 10^00^**
	Mean	5.48 × 10^01^	8.82 × 10^03^	1.88 × 10^04^	0.00 × 10^00^	2.53 × 10^−02^	0.00 × 10^00^	1.57 × 10^−8^	**0.00 × 10^00^**
	Standard	5.89 × 10^01^	4.09 × 10^03^	6.63 × 10^03^	0.00 × 10^00^	1.38 × 10^−11^	0.00 × 10^00^	2.94 × 10^−9^	**0.00 × 10^00^**
	Contest	3.98 × 10^−8^ (+)	7.57 × 10^−12^ (+)	7.57 × 10^−12^ (+)	2.63 × 10^−8^ (+)	7.46 × 10^−12^ (+)	3.43 × 10^−02^ (+)	7.57 × 10^−12^ (+)	
F2(x)	Best	2.28 × 10^01^	4.91 × 10^01^	8.97 × 10^01^	2.66 × 10^−11^	2.39 × 10^−8^	2.32 × 10^−04^	2.47 × 10^−02^	6.67 × 10^00^
	Worst	7.85 × 10^01^	7.43 × 10^01^	4.98 × 10^02^	6.73 × 10^01^	6.66 × 10^01^	7.15 × 10^01^	7.07 × 10^01^	**4.91 × 10^01^**
	Median	5.94 × 10^01^	4.91 × 10^01^	1.62 × 10^02^	4.91 × 10^01^	4.91 × 10^01^	5.14 × 10^01^	4.70 × 10^01^	4.91 × 10^01^
	Mean	5.86 × 10^01^	5.68 × 10^01^	1.96 × 10^02^	4.45 × 10^01^	4.15 × 10^01^	5.09 × 10^01^	3.12 × 10^01^	4.54 × 10^01^
	Standard	1.07 × 10^01^	1.11 × 10^01^	1.05 × 10^02^	1.71 × 10^01^	1.88 × 10^01^	1.50 × 10^01^	2.55 × 10^01^	**8.82 × 10^00^**
	Contest	5.04 × 10^−10^ (+)	3.00 × 10^−11^ (+)	3.00 × 10^−11^ (+)	8.19 × 10^−11^ (=)	4.97 × 10^−03^ (−)	8.44 × 10^−9^ (+)	7.51 × 10^−11^ (=)	
F3(x)	Best	0.00 × 10^00^	9.09 × 10^−02^	1.02 × 10^01^	0.00 × 10^00^	3.19 × 10^−07^	0.00 × 10^00^	6.42 × 10^−11^	7.22 × 10^−04^
	Worst	7.91 × 10^−02^	4.40 × 10^−11^	3.43 × 10^01^	9.10 × 10^−02^	5.53 × 10^−03^	5.94 × 10^−11^	1.85 × 10^01^	8.08 × 10^−11^
	Median	1.14 × 10^−13^	1.82 × 10^−11^	1.75 × 10^01^	0.00 × 10^00^	1.69 × 10^−05^	8.24 × 10^−03^	4.70 × 10^00^	6.66 × 10^−02^
	Mean	1.21 × 10^−02^	2.02 × 10^−11^	1.83 × 10^01^	3.03 × 10^−03^	4.48 × 10^−04^	1.02 × 10^−11^	7.11 × 10^00^	1.28 × 10^−11^
	Standard	2.06 × 10^−02^	8.29 × 10^−02^	6.23 × 10^00^	1.66 × 10^−02^	1.21 × 10^−03^	1.68 × 10^−11^	6.15 × 10^00^	1.71 × 10^−11^
	Contest	1.17 × 10^−06^ (−)	9.52 × 10^−04^ (+)	3.02 × 10^−11^ (+)	1.37 × 10^−11^ (−)	5.46 × 10^−11^ (−)	2.71 × 10^−02^ (−)	4.08 × 10^−11^ (+)	
F4(X)	Best	8.33 × 10^00^	1.81 × 10^01^	3.08 × 10^01^	1.69 × 10^01^	5.97 × 10^00^	5.97 × 10^00^	3.28 × 10^01^	6.96 × 10^00^
	Worst	6.07 × 10^01^	1.14 × 10^02^	6.69 × 10^01^	5.27 × 10^01^	1.49 × 10^01^	4.38 × 10^01^	9.15 × 10^01^	1.99 × 10^01^
	Median	3.89 × 10^01^	4.33 × 10^01^	4.98 × 10^01^	3.34 × 10^01^	8.95 × 10^00^	1.99 × 10^01^	4.97 × 10^01^	1.69 × 10^01^
	Mean	4.01 × 10^01^	4.71 × 10^01^	5.00 × 10^01^	3.20 × 10^01^	9.42 × 10^00^	2.07 × 10^01^	5.38 × 10^01^	1.62 × 10^01^
	Standard	1.08 × 10^01^	1.75 × 10^01^	9.60 × 10^00^	1.08 × 10^01^	2.66 × 10^00^	8.06 × 10^00^	1.54 × 10^01^	3.27 × 10^00^
	Contest	4.48 × 10^−10^ (+)	5.31 × 10^−11^ (+)	2.92 × 10^−11^ (+)	5.55 × 10^−10^ (+)	1.91 × 10^−8^ (−)	2.78 × 10^−02^ (+)	2.92 × 10^−11^ (+)	
F5(X)	Best	1.77 × 10^−07^	2.37 × 10^−02^	5.38 × 10^02^	0.00 × 10^00^	0.00 × 10^00^	0.00 × 10^00^	3.51 × 10^−9^	**0.00 × 10^00^**
	Worst	1.56 × 10^00^	2.64 × 10^00^	2.93 × 10^03^	3.34 × 10^01^	3.97 × 10^00^	1.29 × 10^01^	8.87 × 10^02^	**0.00 × 10^00^**
	Median	5.08 × 10^−11^	2.00 × 10^−11^	1.33 × 10^03^	2.24 × 10^00^	4.48 × 10^−02^	1.14 × 10^−13^	9.09 × 10^−11^	**0.00 × 10^00^**
	Mean	6.13 × 10^−11^	4.13 × 10^−11^	1.41 × 10^03^	5.77 × 10^00^	4.27 × 10^−11^	1.11 × 10^00^	3.98 × 10^01^	**0.00 × 10^00^**
	Standard	4.49 × 10^−11^	5.54 × 10^−11^	5.85 × 10^02^	8.84 × 10^00^	8.98 × 10^−11^	2.89 × 10^00^	1.62 × 10^02^	**0.00 × 10^00^**
	Contest	1.40 × 10^−11^ (+)	1.40 × 10^−11^ (+)	1.40 × 10^−11^ (+)	7.34 × 10^−11^ (+)	4.94 × 10^−11^ (=)	8.70 × 10^−11^ (=)	1.40 × 10^−11^ (+)	
F6(X)	Best	1.86 × 10^02^	2.53 × 10^02^	2.78 × 10^−14^	3.82 × 10^01^	6.54 × 10^01^	2.16 × 10^01^	1.44 × 10^02^	5.55 × 10^01^
	Worst	3.29 × 10^03^	2.17 × 10^04^	6.58E+08	4.52 × 10^03^	6.74 × 10^03^	7.07 × 10^03^	2.30 × 10^04^	**2.47 × 10^02^**
	Median	8.08 × 10^02^	3.82 × 10^03^	2.79E+06	7.34 × 10^02^	1.49 × 10^03^	3.14 × 10^02^	1.95 × 10^03^	**1.26 × 10^02^**
	Mean	1.03 × 10^03^	5.20 × 10^03^	4.94E+07	1.29 × 10^03^	2.01 × 10^03^	1.65 × 10^03^	3.46 × 10^03^	**1.30 × 10^02^**
	Standard	8.26 × 10^02^	5.68 × 10^03^	1.41E+08	1.21 × 10^03^	1.76 × 10^03^	2.31 × 10^03^	5.12 × 10^03^	**5.08 × 10^01^**
	Contest	6.70 × 10^−11^ (+)	3.02 × 10^−11^ (+)	3.02 × 10^−11^ (+)	9.26 × 10^−9^ (+)	7.77 × 10^−9^ (+)	2.07 × 10^−02^ (+)	1.17 × 10^−9^ (+)	
F7(X)	Best	2.13 × 10^01^	1.82 × 10^01^	5.92 × 10^01^	2.19 × 10^01^	2.31 × 10^01^	2.63 × 10^01^	2.20 × 10^01^	2.11 × 10^01^
	Worst	5.24 × 10^01^	9.03 × 10^01^	2.05 × 10^02^	6.74 × 10^01^	7.53 × 10^01^	5.35 × 10^01^	8.82 × 10^01^	3.00 × 10^01^
	Median	3.82 × 10^01^	4.03 × 10^01^	1.14 × 10^02^	3.70 × 10^01^	3.87 × 10^01^	3.28 × 10^01^	3.74 × 10^01^	2.66 × 10^01^
	Mean	3.67 × 10^01^	4.49 × 10^01^	1.20 × 10^02^	3.81 × 10^01^	4.16 × 10^01^	3.58 × 10^01^	4.16 × 10^01^	2.58 × 10^01^
	Standard	7.47 × 10^00^	1.79 × 10^01^	3.97 × 10^01^	1.24 × 10^01^	1.31 × 10^01^	7.73 × 10^00^	1.51 × 10^01^	**2.75 × 10^00^**
	Contest	3.01 × 10^−07^ (−)	6.01 × 10^−8^ (+)	3.02 × 10^−11^ (+)	1.53 × 10^−05^ (+)	5.00 × 10^−9^ (+)	1.31 × 10^−8^ (+)	2.60 × 10^−8^ (+)	
F8(X)	Best	2.16 × 10^01^	2.18 × 10^01^	2.96 × 10^01^	2.04 × 10^01^	2.04 × 10^01^	2.06 × 10^01^	2.34 × 10^01^	**1.90 × 10^01^**
	Worst	3.17 × 10^01^	2.62 × 10^02^	3.25 × 10^02^	1.21 × 10^02^	1.42 × 10^02^	2.51 × 10^01^	4.13 × 10^01^	2.91 × 10^01^
	Median	2.70 × 10^01^	1.44 × 10^02^	3.88 × 10^01^	2.12 × 10^01^	2.13 × 10^01^	2.18 × 10^01^	2.62 × 10^01^	2.40 × 10^01^
	Mean	2.70 × 10^01^	1.07 × 10^02^	7.49 × 10^01^	2.47 × 10^01^	2.92 × 10^01^	2.21 × 10^01^	2.69 × 10^01^	2.46 × 10^01^
	Standard	2.36 × 10^00^	7.85 × 10^01^	8.19 × 10^01^	1.82 × 10^01^	3.04 × 10^01^	1.08 × 10^00^	3.31 × 10^00^	2.60 × 10^00^
	Contest	7.70 × 10^−04^ (+)	1.29 × 10^−06^ (+)	3.02 × 10^−11^ (+)	6.05 × 10^−07^ (+)	3.83 × 10^−06^ (+)	4.08 × 10^−05^ (−)	2.89 × 10^−03^ (+)	
F9(X)	Best	1.83 × 10^02^	1.81 × 10^02^	2.35 × 10^02^	1.81 × 10^02^	1.81 × 10^02^	1.83 × 10^02^	1.81 × 10^02^	1.82 × 10^02^
	Worst	1.84 × 10^02^	1.81 × 10^02^	5.39 × 10^02^	1.81 × 10^02^	1.81 × 10^02^	1.89 × 10^02^	1.81 × 10^02^	1.85 × 10^02^
	Median	1.84 × 10^02^	1.81 × 10^02^	3.90 × 10^02^	1.81 × 10^02^	1.81 × 10^02^	1.85 × 10^02^	1.81 × 10^02^	1.84 × 10^02^
	Mean	1.84 × 10^02^	1.81 × 10^02^	3.83 × 10^02^	1.81 × 10^02^	1.81 × 10^02^	1.85 × 10^02^	1.81 × 10^02^	1.84 × 10^02^
	Standard	4.88 × 10^−11^	1.60 × 10^−02^	7.32 × 10^01^	0.00 × 10^00^	4.51 × 10^−05^	1.67 × 10^00^	2.48 × 10^−05^	7.37 × 10^−11^
	Contest	4.20 × 10^−11^ (=)	3.02 × 10^−11^ (−)	3.02 × 10^−11^ (+)	2.36 × 10^−12^ (−)	3.01 × 10^−11^ (−)	5.32 × 10^−03^ (+)	3.02 × 10^−11^ (−)	
F10(X)	Best	6.60 × 10^01^	2.16 × 10^02^	1.01 × 10^02^	1.27 × 10^01^	1.00 × 10^02^	1.00 × 10^02^	1.00 × 10^02^	1.00 × 10^02^
	Worst	1.24 × 10^02^	2.61 × 10^02^	2.80 × 10^03^	4.98 × 10^02^	8.76 × 10^02^	2.78 × 10^02^	1.71 × 10^03^	**1.01 × 10^02^**
	Median	1.06 × 10^02^	2.39 × 10^02^	1.13 × 10^02^	1.01 × 10^02^	1.01 × 10^02^	1.00 × 10^02^	1.00 × 10^02^	**1.00 × 10^02^**
	Mean	1.06 × 10^02^	2.39 × 10^02^	4.53 × 10^02^	1.80 × 10^02^	1.87 × 10^02^	1.35 × 10^02^	1.85 × 10^02^	**1.00 × 10^02^**
	Standard	1.01 × 10^01^	1.10 × 10^01^	7.57 × 10^02^	1.21 × 10^02^	1.76 × 10^02^	6.39 × 10^01^	2.97 × 10^02^	8.67 × 10^−02^
	Contest	4.80 × 10^−07^ (+)	3.02 × 10^−11^ (+)	3.02 × 10^−11^ (+)	2.43 × 10^−05^ (+)	5.00 × 10^−9^ (+)	1.05 × 10^−11^ (=)	7.06 × 10^−11^ (=)	
F11(X)	Best	5.60 × 10^−11^	3.01 × 10^02^	8.19 × 10^02^	3.00 × 10^02^	3.00 × 10^02^	0.00 × 10^00^	2.67 × 10^−03^	**0.00 × 10^00^**
	Worst	5.43 × 10^02^	4.02 × 10^02^	4.10 × 10^03^	9.06 × 10^02^	4.00 × 10^02^	4.00 × 10^02^	1.76 × 10^03^	**3.00 × 10^02^**
	Median	3.00 × 10^02^	3.02 × 10^02^	1.60 × 10^03^	3.00 × 10^02^	3.00 × 10^02^	3.00 × 10^02^	3.00 × 10^02^	3.00 × 10^02^
	Mean	3.21 × 10^02^	3.09 × 10^02^	1.91 × 10^03^	4.29 × 10^02^	3.10 × 10^02^	3.13 × 10^02^	3.86 × 10^02^	2.80 × 10^02^
	Standard	1.23 × 10^02^	2.52 × 10^01^	9.73 × 10^02^	2.09 × 10^02^	3.05 × 10^01^	7.30 × 10^01^	3.32 × 10^02^	7.61 × 10^01^
	Contest	5.21 × 10^−04^ (+)	4.11 × 10^−12^ (+)	4.11 × 10^−12^ (+)	4.08 × 10^−12^ (+)	2.38 × 10^−11^ (=)	5.64 × 10^−02^ (=)	1.28 × 10^−9^ (+)	
F12(X)	Best	2.42 × 10^02^	2.36 × 10^02^	2.65 × 10^02^	2.34 × 10^02^	2.37 × 10^02^	2.47 × 10^02^	2.36 × 10^02^	2.53 × 10^02^
	Worst	3.00 × 10^02^	2.73 × 10^02^	5.30 × 10^02^	2.95 × 10^02^	2.76 × 10^02^	3.50 × 10^02^	5.03 × 10^02^	2.70 × 10^02^
	Median	2.69 × 10^02^	2.44 × 10^02^	3.19 × 10^02^	2.50 × 10^02^	2.53 × 10^02^	2.75 × 10^02^	2.52 × 10^02^	2.62 × 10^02^
	Mean	2.69 × 10^02^	2.48 × 10^02^	3.39 × 10^02^	2.53 × 10^02^	2.55 × 10^02^	2.77 × 10^02^	2.67 × 10^02^	2.62 × 10^02^
	Standard	1.22 × 10^01^	9.10 × 10^00^	6.34 × 10^01^	1.35 × 10^01^	1.10 × 10^01^	2.22 × 10^01^	4.92 × 10^01^	3.94 × 10^00^
	Contest	3.85 × 10^−03^ (+)	3.65 × 10^−8^ (−)	7.39 × 10^−11^ (+)	7.70 × 10^−04^ (−)	1.52 × 10^−03^ (−)	4.98 × 10^−04^ (+)	1.70 × 10^−02^ (+)	
+/=/−		2009/1/2	10/0/2	12/0/0	2008/1/3	2005/2/5	2007/3/2	2009/2/1	
Rank		4.42	5.12	7.83	3.50	3.58	3.92	5.00	2.58

**Table 3:** Ten dimensions with other algorithms.

F	Index	APGSK−IMODE	EA4eig	IMODE	PVADE	AGSK	RPSO
F1(x)	Best	0.00 × 10^00^	0.00 × 10^00^	0.00 × 10^00^	0.00 × 10^00^	0.00 × 10^00^	**0.00 × 10^00^**
	Worst	0.00 × 10^00^	0.00 × 10^00^	0.00 × 10^00^	0.00 × 10^00^	0.00 × 10^00^	**0.00 × 10^00^**
	Median	0.00 × 10^00^	0.00 × 10^00^	0.00 × 10^00^	0.00 × 10^00^	0.00 × 10^00^	**0.00 × 10^00^**
	Mean	0.00 × 10^00^	0.00 × 10^00^	0.00 × 10^00^	0.00 × 10^00^	0.00 × 10^00^	**0.00 × 10^00^**
	Standard	0.00 × 10^00^	0.00 × 10^00^	0.00 × 10^00^	0.00 × 10^00^	0.00 × 10^00^	**0.00 × 10^00^**
	Contest	N/A (=)	N/A (=)	N/A (=)	N/A (=)	N/A (=)	
F2(x)	Best	0.00 × 10^00^	0.00 × 10^00^	0.00 × 10^00^	0.00 × 10^00^	0.00 × 10^00^	1.71 × 10^−07^
	Worst	0.00 × 10^00^	3.99 × 10^00^	0.00 × 10^00^	8.92 × 10^00^	8.92 × 10^00^	7.32 × 10^−02^
	Median	0.00 × 10^00^	0.00 × 10^00^	0.00 × 10^00^	8.92 × 10^00^	3.99 × 10^00^	5.91 × 10^−04^
	Mean	0.00 × 10^00^	6.64 × 10^−11^	0.00 × 10^00^	5.55 × 10^00^	3.09 × 10^00^	9.12 × 10^−03^
	Standard	0.00 × 10^00^	1.51 × 10^00^	0.00 × 10^00^	3.91 × 10^00^	2.09 × 10^00^	1.80 × 10^−02^
	Contest	1.21 × 10^−12^ (−)	4.70 × 10^−06^ (+)	1.21 × 10^−12^ (−)	1.74 × 10^−03^ (+)	1.53 × 10^−03^ (+)	
F3(x)	Best	0.00 × 10^00^	0.00 × 10^00^	2.69 × 10^−07^	0.00 × 10^00^	0.00 × 10^00^	**0.00 × 10^00^**
	Worst	0.00 × 10^00^	0.00 × 10^00^	4.86 × 10^−05^	4.85 × 10^−05^	2.08 × 10^−05^	8.79 × 10^−05^
	Median	0.00 × 10^00^	0.00 × 10^00^	1.73 × 10^−06^	0.00 × 10^00^	1.42 × 10^−06^	1.42 × 10^−06^
	Mean	0.00 × 10^00^	0.00 × 10^00^	5.93 × 10^−06^	1.93 × 10^−06^	2.75 × 10^−06^	7.62 × 10^−06^
	Standard	0.00 × 10^00^	0.00 × 10^00^	9.55 × 10^−06^	8.84 × 10^−06^	4.75 × 10^−06^	1.90 × 10^−05^
	Contest	9.65 × 10^−11^ (−)	5.63 × 10^−11^ (−)	8.75 × 10^−02^ (=)	1.98 × 10^−06^ (−)	1.62 × 10^−11^ (=)	
F4(X)	Best	9.96 × 10^−11^	0.00 × 10^00^	4.97 × 10^00^	2.72 × 10^00^	3.02 × 10^00^	2.98 × 10^00^
	Worst	6.96 × 10^00^	2.98 × 10^00^	1.29 × 10^01^	8.68 × 10^00^	1.13 × 10^01^	9.95 × 10^00^
	Median	4.97 × 10^00^	9.95 × 10^−11^	8.95 × 10^00^	5.48 × 10^00^	7.97 × 10^00^	8.46 × 10^00^
	Mean	4.61 × 10^00^	9.62 × 10^−11^	9.02 × 10^00^	5.45 × 10^00^	7.63 × 10^00^	7.63 × 10^00^
	Standard	1.21 × 10^00^	9.23 × 10^−11^	2.20 × 10^00^	1.65 × 10^00^	2.14 × 10^00^	2.08 × 10^00^
	Contest	7.86 × 10^−06^ (−)	2.39 × 10^−11^ (−)	2.06 × 10^−02^ (+)	9.69 × 10^−05^ (−)	9.06 × 10^−11^ (=)	
F5(X)	Best	0.00 × 10^00^	0.00 × 10^00^	0.00 × 10^00^	0.00 × 10^00^	0.00 × 10^00^	**0.00 × 10^00^**
	Worst	0.00 × 10^00^	0.00 × 10^00^	1.68 × 10^01^	0.00 × 10^00^	1.14 × 10^−13^	**0.00 × 10^00^**
	Median	0.00 × 10^00^	0.00 × 10^00^	6.33 × 10^−11^	0.00 × 10^00^	0.00 × 10^00^	**0.00 × 10^00^**
	Mean	0.00 × 10^00^	0.00 × 10^00^	1.83 × 10^00^	0.00 × 10^00^	7.58 × 10^−15^	**0.00 × 10^00^**
	Standard	0.00 × 10^00^	0.00 × 10^00^	3.20 × 10^00^	0.00 × 10^00^	2.88 × 10^−14^	**0.00 × 10^00^**
	Contest	N/A (=)	N/A (=)	6.93 × 10^−12^ (+)	N/A (=)	5.70 × 10^−11^ (=)	
F6(X)	Best	6.55 × 10^−02^	1.63 × 10^−03^	9.90 × 10^−02^	2.67 × 10^−02^	5.02 × 10^−03^	2.70 × 10^00^
	Worst	4.49 × 10^−11^	4.28 × 10^−11^	4.99 × 10^00^	1.70 × 10^01^	4.75 × 10^−11^	1.96 × 10^02^
	Median	1.86 × 10^−11^	1.85 × 10^−02^	1.18 × 10^00^	3.72 × 10^−11^	2.47 × 10^−11^	6.32 × 10^01^
	Mean	2.30 × 10^−11^	5.70 × 10^−02^	1.65 × 10^00^	1.48 × 10^00^	2.50 × 10^−11^	7.83 × 10^01^
	Standard	1.17 × 10^−11^	9.46 × 10^−02^	1.29 × 10^00^	3.57 × 10^00^	1.32 × 10^−11^	6.02 × 10^01^
	Contest	3.01 × 10^−11^ (−)	3.02 × 10^−11^ (−)	4.98 × 10^−11^ (−)	6.70 × 10^−11^ (−)	3.02 × 10^−11^ (−)	
F7(X)	Best	0.00 × 10^00^	0.00 × 10^00^	2.65 × 10^−05^	0.00 × 10^00^	0.00 × 10^00^	1.96 × 10^−11^
	Worst	1.94 × 10^−07^	3.76 × 10^−9^	1.24 × 10^−03^	2.10 × 10^01^	2.09 × 10^−10^	2.26 × 10^01^
	Median	0.00 × 10^00^	5.68 × 10^−13^	1.71 × 10^−04^	3.12 × 10^−11^	4.55 × 10^−13^	5.20 × 10^00^
	Mean	6.47 × 10^−9^	2.77 × 10^−10^	3.05 × 10^−04^	3.76 × 10^00^	1.83 × 10^−11^	9.88 × 10^00^
	Standard	3.54 × 10^−8^	8.53 × 10^−10^	3.52 × 10^−04^	7.67 × 10^00^	5.06 × 10^−11^	9.20 × 10^00^
	Contest	1.65 × 10^−11^ (−)	2.71 × 10^−11^ (−)	3.02 × 10^−11^ (−)	7.49 × 10^−06^ (−)	2.70 × 10^−11^ (−)	
F8(X)	Best	2.17 × 10^−02^	1.39 × 10^−03^	4.30 × 10^−02^	7.44 × 10^−11^	1.19 × 10^−11^	2.21 × 10^−11^
	Worst	2.64 × 10^00^	8.21 × 10^−11^	8.09 × 10^00^	2.16 × 10^01^	1.61 × 10^00^	2.10 × 10^01^
	Median	1.99 × 10^−11^	3.37 × 10^−11^	1.42 × 10^00^	1.17 × 10^00^	6.47 × 10^−11^	4.36 × 10^00^
	Mean	3.68 × 10^−11^	3.24 × 10^−11^	2.72 × 10^00^	4.35 × 10^00^	7.84 × 10^−11^	6.25 × 10^00^
	Standard	5.11 × 10^−11^	2.21 × 10^−11^	2.34 × 10^00^	7.15 × 10^00^	4.37 × 10^−11^	6.40 × 10^00^
	Contest	2.66 × 10^−9^ (−)	2.44 × 10^−9^ (−)	2.32 × 10^−02^ (−)	4.06 × 10^−02^ (−)	1.03 × 10^−06^ (−)	
F9(X)	Best	2.29 × 10^02^	1.86 × 10^02^	1.37 × 10^−06^	2.29 × 10^02^	2.29 × 10^02^	2.29 × 10^02^
	Worst	2.29 × 10^02^	1.86 × 10^02^	2.29 × 10^02^	2.29 × 10^02^	2.29 × 10^02^	2.29 × 10^02^
	Median	2.29 × 10^02^	1.86 × 10^02^	2.29 × 10^02^	2.29 × 10^02^	2.29 × 10^02^	2.29 × 10^02^
	Mean	2.29 × 10^02^	1.86 × 10^02^	2.14 × 10^02^	2.29 × 10^02^	2.29 × 10^02^	2.29 × 10^02^
	Standard	0.00 × 10^00^	4.22 × 10^−13^	5.82 × 10^01^	0.00 × 10^00^	8.67 × 10^−14^	**0.00 × 10^00^**
	Contest	N/A (=)	4.16 × 10^−14^ (−)	7.64 × 10^−9^ (−)	1.69 × 10^−14^ (−)	N/A (=)	
F10(X)	Best	6.72 × 10^01^	1.00 × 10^02^	3.75 × 10^00^	1.00 × 10^02^	1.00 × 10^02^	1.00 × 10^02^
	Worst	1.00 × 10^02^	1.00 × 10^02^	1.00 × 10^02^	1.00 × 10^02^	1.00 × 10^02^	1.00 × 10^02^
	Median	1.00 × 10^02^	1.00 × 10^02^	1.00 × 10^02^	1.00 × 10^02^	1.00 × 10^02^	1.00 × 10^02^
	Mean	9.91 × 10^01^	1.00 × 10^02^	8.35 × 10^01^	1.00 × 10^02^	1.00 × 10^02^	1.00 × 10^02^
	Standard	6.02 × 10^00^	3.69 × 10^−02^	3.45 × 10^01^	3.82 × 10^−02^	3.49 × 10^−02^	5.59 × 10^−02^
	Contest	9.47 × 10^−11^ (=)	1.24 × 10^−03^ (−)	1.63 × 10^−02^ (−)	6.10 × 10^−03^ (−)	4.20 × 10^−11^ (=)	
F11(X)	Best	0.00 × 10^00^	0.00 × 10^00^	0.00 × 10^00^	0.00 × 10^00^	0.00 × 10^00^	**0.00 × 10^00^**
	Worst	4.55 × 10^−13^	0.00 × 10^00^	2.61 × 10^−07^	3.00 × 10^02^	4.55 × 10^−13^	**0.00 × 10^00^**
	Median	0.00 × 10^00^	0.00 × 10^00^	2.96 × 10^−8^	0.00 × 10^00^	4.55 × 10^−13^	**0.00 × 10^00^**
	Mean	1.36 × 10^−13^	0.00 × 10^00^	5.19 × 10^−8^	1.00 × 10^01^	3.49 × 10^−13^	**0.00 × 10^00^**
	Standard	2.12 × 10^−13^	0.00 × 10^00^	6.11 × 10^−8^	5.48 × 10^01^	1.96 × 10^−13^	**0.00 × 10^00^**
	Contest	5.19 × 10^−02^ (=)	N/A (=)	6.92 × 10^−8^ (+)	4.76 × 10^−05^ (+)	1.56 × 10^−11^ (=)	
F12(X)	Best	1.59 × 10^02^	1.45 × 10^02^	1.63 × 10^02^	1.59 × 10^02^	1.59 × 10^02^	1.65 × 10^02^
	Worst	1.63 × 10^02^	1.59 × 10^02^	1.65 × 10^02^	1.65 × 10^02^	1.61 × 10^02^	1.68 × 10^02^
	Median	1.62 × 10^02^	1.46 × 10^02^	1.64 × 10^02^	1.63 × 10^02^	1.60 × 10^02^	1.66 × 10^02^
	Mean	1.61 × 10^02^	1.47 × 10^02^	1.64 × 10^02^	1.63 × 10^02^	1.60 × 10^02^	1.66 × 10^02^
	Standard	1.54 × 10^00^	3.55 × 10^00^	6.54 × 10^−11^	2.08 × 10^00^	9.91 × 10^−11^	8.15 × 10^−11^
	Contest	2.52 × 10^−11^ (−)	3.02 × 10^−11^ (−)	4.08 × 10^−11^ (−)	9.36 × 10^−11^ (−)	2.56 × 10^−11^ (−)	
+/=/−		2000/5/7	2001/3/8	2003/2/7	2002/2/8	2001/7/4	
Rank		2.54	1.96	4.00	4.12	3.83	4.50

**Table 4:** Twenty dimensions with other algorithms.

F	Index	APGSK^−^IMODE	EA4eig	IMODE	PVADE	AGSK	RPSO
F1(x)	Best	0.00 × 10^00^	0.00 × 10^00^	0.00 × 10^00^	0.00 × 10^00^	0.00 × 10^00^	**0.00 × 10^00^**
	Worst	5.68 × 10^−14^	0.00 × 10^00^	0.00 × 10^00^	0.00 × 10^00^	5.68 × 10^−14^	**0.00 × 10^00^**
	Median	0.00 × 10^00^	0.00 × 10^00^	0.00 × 10^00^	0.00 × 10^00^	0.00 × 10^00^	**0.00 × 10^00^**
	Mean	7.58 × 10^−15^	0.00 × 10^00^	0.00 × 10^00^	0.00 × 10^00^	1.89 × 10^−14^	**0.00 × 10^00^**
	Standard	1.97 × 10^−14^	0.00 × 10^00^	0.00 × 10^00^	0.00 × 10^00^	2.73 × 10^−14^	**0.00 × 10^00^**
	Contest	N/A (=)	N/A (=)	N/A (=)	N/A (=)	N/A (=)	
F2(x)	Best	3.63 × 10^−06^	0.00 × 10^00^	0.00 × 10^00^	0.00 × 10^00^	0.00 × 10^00^	6.67 × 10^00^
	Worst	4.91 × 10^01^	3.99 × 10^00^	4.91 × 10^01^	8.92 × 10^00^	4.91 × 10^01^	4.91 × 10^01^
	Median	4.91 × 10^01^	0.00 × 10^00^	4.91 × 10^01^	3.99 × 10^00^	4.91 × 10^01^	4.91 × 10^01^
	Mean	4.47 × 10^01^	7.97 × 10^−11^	3.45 × 10^01^	5.36 × 10^00^	2.92 × 10^01^	4.54 × 10^01^
	Standard	1.18 × 10^01^	1.62 × 10^00^	2.27 × 10^01^	3.69 × 10^00^	2.42 × 10^01^	8.82 × 10^00^
	Contest	1.20 × 10^−03^ (−)	6.27 × 10^−12^ (−)	3.14 × 10^−11^ (=)	8.20 × 10^−11^ (−)	2.95 × 10^−05^ (−)	
F3(x)	Best	0.00 × 10^00^	0.00 × 10^00^	2.11 × 10^−11^	0.00 × 10^00^	0.00 × 10^00^	3.49 × 10^−03^
	Worst	1.14 × 10^−13^	1.14 × 10^−13^	3.49 × 10^00^	1.42 × 10^−06^	8.36 × 10^−11^	4.56 × 10^00^
	Median	1.14 × 10^−13^	1.14 × 10^−13^	1.03 × 10^00^	0.00 × 10^00^	3.41 × 10^−13^	1.61 × 10^00^
	Mean	1.06 × 10^−13^	7.96 × 10^−14^	1.08 × 10^00^	7.11 × 10^−8^	3.56 × 10^−12^	1.75 × 10^00^
	Standard	2.88 × 10^−14^	5.30 × 10^−14^	6.96 × 10^−11^	2.70 × 10^−07^	1.52 × 10^−11^	1.30 × 10^00^
	Contest	2.36 × 10^−12^ (−)	1.01 × 10^−11^ (−)	2.37 × 10^−10^ (−)	3.16 × 10^−12^ (−)	2.89 × 10^−11^ (−)	
F4(X)	Best	1.29 × 10^01^	3.98 × 10^00^	3.68 × 10^01^	0.00 × 10^00^	1.98 × 10^01^	6.96 × 10^00^
	Worst	2.98 × 10^01^	1.69 × 10^01^	8.36 × 10^01^	5.97 × 10^00^	4.58 × 10^01^	1.99 × 10^01^
	Median	2.19 × 10^01^	8.95 × 10^00^	5.62 × 10^01^	1.99 × 10^00^	3.72 × 10^01^	1.69 × 10^01^
	Mean	2.23 × 10^01^	9.22 × 10^00^	5.86 × 10^01^	2.46 × 10^00^	3.63 × 10^01^	1.62 × 10^01^
	Standard	4.51 × 10^00^	3.49 × 10^00^	1.23 × 10^01^	1.32 × 10^00^	6.85 × 10^00^	3.27 × 10^00^
	Contest	2.58 × 10^−06^ (+)	4.59 × 10^−8^ (−)	2.92 × 10^−11^ (+)	2.58 × 10^−11^ (−)	3.56 × 10^−11^ (+)	
F5(X)	Best	0.00 × 10^00^	0.00 × 10^00^	2.61 × 10^02^	0.00 × 10^00^	1.14 × 10^−13^	**0.00 × 10^00^**
	Worst	0.00 × 10^00^	0.00 × 10^00^	1.03 × 10^03^	0.00 × 10^00^	5.44 × 10^−11^	**0.00 × 10^00^**
	Median	0.00 × 10^00^	0.00 × 10^00^	6.72 × 10^02^	0.00 × 10^00^	8.95 × 10^−02^	**0.00 × 10^00^**
	Mean	0.00 × 10^00^	0.00 × 10^00^	6.82 × 10^02^	0.00 × 10^00^	7.48 × 10^−02^	**0.00 × 10^00^**
	Standard	0.00 × 10^00^	0.00 × 10^00^	2.08 × 10^02^	0.00 × 10^00^	1.11 × 10^−11^	**0.00 × 10^00^**
	Contest	N/A (=)	N/A (=)	1.40 × 10^−11^ (+)	N/A (=)	1.53 × 10^−9^ (+)	
F6(X)	Best	1.04 × 10^00^	2.18 × 10^−02^	9.17 × 10^00^	2.20 × 10^−02^	2.18 × 10^−11^	5.55 × 10^01^
	Worst	2.48 × 10^01^	2.08 × 10^00^	4.54 × 10^01^	1.12 × 10^00^	1.90 × 10^00^	2.47 × 10^02^
	Median	6.91 × 10^00^	1.20 × 10^−11^	2.19 × 10^01^	1.25 × 10^−11^	3.95 × 10^−11^	1.26 × 10^02^
	Mean	9.11 × 10^00^	2.13 × 10^−11^	2.36 × 10^01^	3.23 × 10^−11^	6.36 × 10^−11^	1.30 × 10^02^
	Standard	6.77 × 10^00^	3.69 × 10^−11^	8.37 × 10^00^	3.73 × 10^−11^	5.27 × 10^−11^	5.08 × 10^01^
	Contest	3.01 × 10^−11^ (−)	3.02 × 10^−11^ (−)	3.02 × 10^−11^ (−)	3.02 × 10^−11^ (−)	3.02 × 10^−11^ (−)	
F7(X)	Best	2.51 × 10^−02^	0.00 × 10^00^	2.51 × 10^01^	0.00 × 10^00^	3.12 × 10^−11^	2.11 × 10^01^
	Worst	2.10 × 10^01^	2.10 × 10^01^	4.48 × 10^01^	2.10 × 10^01^	2.25 × 10^01^	3.00 × 10^01^
	Median	3.86 × 10^00^	1.80 × 10^00^	3.36 × 10^01^	0.00 × 10^00^	2.11 × 10^01^	2.66 × 10^01^
	Mean	7.00 × 10^00^	4.23 × 10^00^	3.36 × 10^01^	3.68 × 10^00^	1.82 × 10^01^	2.58 × 10^01^
	Standard	7.08 × 10^00^	6.18 × 10^00^	5.16 × 10^00^	7.62 × 10^00^	6.09 × 10^00^	2.75 × 10^00^
	Contest	3.01 × 10^−11^ (−)	3.00 × 10^−11^ (−)	2.39 × 10^−8^ (−)	1.78 × 10^−11^ (−)	8.89 × 10^−10^ (−)	
F8(X)	Best	1.41 × 10^01^	2.69 × 10^−11^	2.08 × 10^01^	5.64 × 10^−05^	1.61 × 10^01^	1.90 × 10^01^
	Worst	2.09 × 10^01^	2.07 × 10^01^	2.33 × 10^01^	2.04 × 10^01^	2.29 × 10^01^	2.91 × 10^01^
	Median	2.04 × 10^01^	2.02 × 10^01^	2.18 × 10^01^	1.06 × 10^−11^	2.21 × 10^01^	2.40 × 10^01^
	Mean	2.01 × 10^01^	1.77 × 10^01^	2.19 × 10^01^	1.69 × 10^00^	2.20 × 10^01^	2.46 × 10^01^
	Standard	1.44 × 10^00^	6.14 × 10^00^	6.63 × 10^−11^	5.16 × 10^00^	1.16 × 10^00^	2.60 × 10^00^
	Contest	4.61 × 10^−10^ (−)	3.16 × 10^−10^ (−)	3.83 × 10^−06^ (−)	3.69 × 10^−11^ (−)	1.02 × 10^−05^ (−)	
F9(X)	Best	1.81 × 10^02^	1.65 × 10^02^	1.81 × 10^02^	2.29 × 10^02^	1.81 × 10^02^	1.82 × 10^02^
	Worst	1.81 × 10^02^	1.65 × 10^02^	1.81 × 10^02^	2.29 × 10^02^	1.81 × 10^02^	1.85 × 10^02^
	Median	1.81 × 10^02^	1.65 × 10^02^	1.81 × 10^02^	2.29 × 10^02^	1.81 × 10^02^	1.84 × 10^02^
	Mean	1.81 × 10^02^	1.65 × 10^02^	1.81 × 10^02^	2.29 × 10^02^	1.81 × 10^02^	1.84 × 10^02^
	Standard	8.67 × 10^−14^	0.00 × 10^00^	1.18 × 10^−07^	0.00 × 10^00^	8.67 × 10^−14^	7.37 × 10^−11^
	Contest	1.21 × 10^−12^ (−)	1.21 × 10^−12^ (−)	3.02 × 10^−11^ (−)	1.21 × 10^−12^ (+)	1.21 × 10^−12^ (−)	
F10(X)	Best	1.00 × 10^02^	1.00 × 10^02^	1.00 × 10^02^	1.00 × 10^02^	1.00 × 10^02^	1.00 × 10^02^
	Worst	1.00 × 10^02^	2.24 × 10^02^	1.01 × 10^02^	2.00 × 10^02^	1.00 × 10^02^	1.01 × 10^02^
	Median	1.00 × 10^02^	1.00 × 10^02^	1.01 × 10^02^	1.00 × 10^02^	1.00 × 10^02^	1.00 × 10^02^
	Mean	1.00 × 10^02^	1.08 × 10^02^	1.01 × 10^02^	1.03 × 10^02^	1.00 × 10^02^	1.00 × 10^02^
	Standard	3.76 × 10^−02^	3.06 × 10^01^	7.66 × 10^−02^	1.82 × 10^01^	2.85 × 10^−02^	8.67 × 10^−02^
	Contest	9.21 × 10^−05^ (−)	1.01 × 10^−8^ (−)	6.53 × 10^−8^ (+)	5.57 × 10^−10^ (−)	8.99 × 10^−11^ (−)	
F11(X)	Best	0.00 × 10^00^	3.00 × 10^02^	3.35 × 10^−04^	0.00 × 10^00^	3.00 × 10^02^	**0.00 × 10^00^**
	Worst	3.00 × 10^02^	4.00 × 10^02^	3.22 × 10^02^	1.50 × 10^02^	4.00 × 10^02^	3.00 × 10^02^
	Median	3.00 × 10^02^	3.00 × 10^02^	3.00 × 10^02^	0.00 × 10^00^	4.00 × 10^02^	3.00 × 10^02^
	Mean	2.50 × 10^02^	3.23 × 10^02^	2.81 × 10^02^	5.01 × 10^00^	3.87 × 10^02^	2.80 × 10^02^
	Standard	1.14 × 10^02^	4.30 × 10^01^	7.64 × 10^01^	2.75 × 10^01^	3.46 × 10^01^	7.61 × 10^01^
	Contest	1.08 × 10^−11^ (=)	2.18 × 10^−02^ (+)	1.28 × 10^−9^ (+)	6.71 × 10^−13^ (−)	8.54 × 10^−12^ (+)	
F12(X)	Best	2.31 × 10^02^	1.89 × 10^02^	2.40 × 10^02^	1.60 × 10^02^	2.31 × 10^02^	2.53 × 10^02^
	Worst	2.37 × 10^02^	2.00 × 10^02^	2.67 × 10^02^	1.65 × 10^02^	2.39 × 10^02^	2.70 × 10^02^
	Median	2.33 × 10^02^	2.00 × 10^02^	2.53 × 10^02^	1.64 × 10^02^	2.34 × 10^02^	2.62 × 10^02^
	Mean	2.34 × 10^02^	2.00 × 10^02^	2.53 × 10^02^	1.63 × 10^02^	2.34 × 10^02^	2.62 × 10^02^
	Standard	1.52 × 10^00^	2.05 × 10^00^	7.63 × 10^00^	1.51 × 10^00^	2.07 × 10^00^	3.94 × 10^00^
	Contest	3.02 × 10^−11^ (−)	3.02 × 10^−11^ (−)	7.74 × 10^−06^ (−)	2.89 × 10^−11^ (−)	2.97 × 10^−11^ (−)	
+/=/−		2001/3/8	2001/2/9	2004/2/6	2001/2/9	2003/1/8	
Rank		3.21	2.33	4.54	2.42	4.00	4.50

**Table 5:** Data from ablation experiments.

F	Index	Best	Worst	Median	Mean	Standard
F1(x)	PSO^−^1	0.00 × 10^00^	0.00 × 10^00^	0.00 × 10^00^	0.00 × 10^00^	0.00 × 10^00^
	PSO^−^2	0.00 × 10^00^	0.00 × 10^00^	0.00 × 10^00^	0.00 × 10^00^	0.00 × 10^00^
	PSO^−^3	0.00 × 10^00^	0.00 × 10^00^	0.00 × 10^00^	0.00 × 10^00^	0.00 × 10^00^
	RPSO	**0.00 × 10^00^**	**0.00 × 10^00^**	**0.00 × 10^00^**	**0.00 × 10^00^**	**0.00 × 10^00^**
F2(x)	PSO^−^1	0.00 × 10^00^	1.08 × 10^01^	3.99 × 10^00^	5.15 × 10^00^	4.29 × 10^00^
	PSO^−^2	0.00 × 10^00^	1.31 × 10^01^	6.33 × 10^00^	5.97 × 10^00^	4.09 × 10^00^
	PSO^−^3	0.00 × 10^00^	1.04 × 10^01^	3.99 × 10^00^	3.46 × 10^00^	3.53 × 10^00^
	RPSO	1.71 × 10^−07^	**7.32 × 10^−02^**	**5.91** × 10^−04^	**9.12 × 10^−03^**	**1.80 × 10^−02^**
F3(x)	PSO^−^1	0.00 × 10^00^	2.31 × 10^−02^	1.26 × 10^−04^	2.80 × 10^−03^	6.28 × 10^−03^
	PSO^−^2	1.06E^−^10	7.47 × 10^−02^	6.67 × 10^−04^	9.25 × 10^−03^	1.89 × 10^−02^
	PSO^−^3	0.00 × 10^00^	1.91 × 10^−02^	9.10 × 10^−05^	3.05 × 10^−03^	5.55 × 10^−03^
	RPSO	**0.00 × 10^00^**	**8.79 × 10^−05^**	**1.42** × 10^−06^	**7.62** × 10^−06^	**1.90 × 10^−05^**
F4(X)	PSO^−^1	2.98 × 10^00^	2.09 × 10^01^	6.96 × 10^00^	8.76 × 10^00^	4.74 × 10^00^
	PSO^−^2	9.95 × 10^−01^	1.49 × 10^01^	5.97 × 10^00^	7.23 × 10^00^	3.74 × 10^00^
	PSO^−^3	9.95 × 10^−01^	1.69 × 10^01^	7.46 × 10^00^	8.13 × 10^00^	4.27 × 10^00^
	RPSO	2.98 × 10^00^	**9.95 × 10^00^**	8.46 × 10^00^	7.63 × 10^00^	**2.08 × 10^00^**
F5(X)	PSO^−^1	0.00 × 10^00^	4.54 × 10^−01^	0.00 × 10^00^	2.11 × 10^−02^	8.49 × 10^−02^
	PSO^−^2	0.00 × 10^00^	8.95 × 10^−02^	0.00 × 10^00^	2.98 × 10^−03^	1.63 × 10^−02^
	PSO^−^3	0.00 × 10^00^	1.79 × 10^−01^	0.00 × 10^00^	2.09 × 10^−02^	4.51 × 10^−02^
	RPSO	**0.00 × 10^00^**	**0.00 × 10^00^**	**0.00 × 10^00^**	**0.00 × 10^00^**	**0.00 × 10^00^**
F6(X)	PSO^−^1	2.95 × 10^00^	1.72 × 10^−03^	4.73 × 10^01^	2.26 × 10^02^	4.03 × 10^02^
	PSO^−^2	1.28 × 10^00^	2.55 × 10^−03^	3.93 × 10^01^	2.10 × 10^02^	4.89 × 10^02^
	PSO^−^3	1.52 × 10^00^	1.45 × 10^−03^	4.53 × 10^01^	1.90 × 10^02^	3.35 × 10^02^
	RPSO	2.70 × 10^00^	**1.96 × 10^02^**	6.32 × 10^01^	**7.83 × 10^01^**	**6.02 × 10^01^**
F7(X)	PSO^−^1	2.69 × 10^−08^	2.55 × 10^01^	1.28 × 10^01^	1.20 × 10^01^	9.76 × 10^00^
	PSO^−^2	6.61 × 10^−05^	2.30 × 10^01^	5.23 × 10^00^	1.05 × 10^01^	9.26 × 10^00^
	PSO^−^3	8.44E^−^09	2.56 × 10^01^	1.28 × 10^01^	1.19 × 10^01^	1.02 × 10^01^
	RPSO	1.96 × 10^−01^	**2.26 × 10^01^**	**5.20 × 10^00^**	**9.88 × 10^00^**	**9.20 × 10^00^**
F8(X)	PSO^−^1	1.76 × 10^−01^	2.47 × 10^01^	2.06 × 10^01^	1.43 × 10^01^	9.86 × 10^00^
	PSO^−^2	8.83 × 10^−01^	2.29 × 10^01^	2.02 × 10^01^	1.29 × 10^01^	9.29 × 10^00^
	PSO^−^3	2.84 × 10^−01^	2.42 × 10^01^	2.10 × 10^01^	1.36 × 10^01^	9.57 × 10^00^
	RPSO	2.21 × 10^−01^	**2.10 × 10^01^**	**4.36 × 10^00^**	**6.25 × 10^00^**	**6.40 × 10^00^**
F9(X)	PSO^−^1	2.30 × 10^02^	3.76 × 10^02^	2.32 × 10^02^	2.36 × 10^02^	2.64 × 10^01^
	PSO^−^2	2.30 × 10^02^	2.32 × 10^02^	2.31 × 10^02^	2.31 × 10^02^	5.53 × 10^−01^
	PSO^−^3	2.30 × 10^02^	2.32 × 10^02^	2.31 × 10^02^	2.31 × 10^02^	4.77 × 10^−01^
	RPSO	**2.29 × 10^02^**	**2.29 × 10^02^**	**2.29 × 10^02^**	**2.29 × 10^02^**	**0.00 × 10^00^**
F10(X)	PSO^−^1	1.00 × 10^02^	1.22 × 10^02^	1.00 × 10^02^	1.04 × 10^02^	6.10 × 10^00^
	PSO^−^2	1.00 × 10^02^	2.20 × 10^02^	1.00 × 10^02^	1.49 × 10^02^	5.70 × 10^01^
	PSO^−^3	1.00 × 10^02^	2.20 × 10^02^	1.00 × 10^02^	1.45 × 10^02^	5.55 × 10^01^
	RPSO	**1.00 × 10^02^**	**1.00 × 10^02^**	**1.00 × 10^02^**	**1.00 × 10^02^**	**5.59 × 10^−02^**
F11(X)	PSO^−^1	0.00 × 10^00^	3.00 × 10^02^	0.00 × 10^00^	4.01 × 10^01^	8.76 × 10^01^
	PSO^−^2	0.00 × 10^00^	4.00 × 10^02^	0.00 × 10^00^	8.22 × 10^01^	1.20 × 10^02^
	PSO^−^3	0.00 × 10^00^	4.00 × 10^02^	0.00 × 10^00^	4.34 × 10^01^	9.08 × 10^01^
	RPSO	**0.00 × 10^00^**	**0.00 × 10^00^**	**0.00 × 10^00^**	**0.00 × 10^00^**	**0.00 × 10^00^**
F12(X)	PSO^−^1	1.65 × 10^02^	1.67 × 10^02^	1.66 × 10^02^	1.66 × 10^02^	6.60 × 10^−01^
	PSO^−^2	1.64 × 10^02^	1.67 × 10^02^	1.65 × 10^02^	1.66 × 10^02^	7.50 × 10^−01^
	PSO^−^3	1.65 × 10^02^	1.68 × 10^02^	1.65 × 10^02^	1.66 × 10^02^	8.05 × 10^−01^
	RPSO	1.65 × 10^02^	1.68 × 10^02^	1.66 × 10^02^	1.66 × 10^02^	8.15 × 10^−01^

**Table 6:** Classification results for each algorithm.

Method	Dataset	Accuracy	Precision	Recall	F1 Score	Best	Mean	Worst
PSO	XY	98.21%	98.82%	97.67%	98.25%	98.21%	97.86%	97.02%
	CT	87.05%	91.30%	80.00%	85.28%	87.05%	84.38%	83.04%
GWO	XY	99.40%	100.00%	98.84%	99.42%	99.40%	98.10%	96.43%
	CT	86.16%	84.91%	85.71%	85.31%	86.16%	84.91%	83.93%
WOA	XY	98.81%	98.84%	98.84%	98.84%	98.81%	97.62%	95.83%
	CT	86.61%	84.40%	87.62%	85.98%	86.61%	84.91%	83.48%
DEPSO	XY	98.81%	98.84%	98.84%	98.84%	98.81%	98.45%	97.62%
	CT	83.93%	84.85%	80.00%	82.35%	83.93%	82.59%	79.91%
ASSOA	XY	98.81%	100.00%	97.67%	98.82%	98.81%	97.86%	96.43%
	CT	85.71%	85.44%	83.81%	84.62%	85.71%	84.73%	83.93%
RPSO	XY	100%	100%	100%	100%	100%	98.69%	97.02%
	CT	87.05%	88.00%	83.81%	85.85%	87.05%	85.71%	84.82%

## Data Availability

Data are contained within the article.
